# Supplementary figures and images for: Assessing reliability of intra-tumor heterogeneity estimates from single sample whole exome sequencing data
Source: PLoS One. 2019 Nov 7;14(11):e0224143. doi: 10.1371/journal.pone.0224143 (PMC6837753; doi:10.1371/journal.pone.0224143)

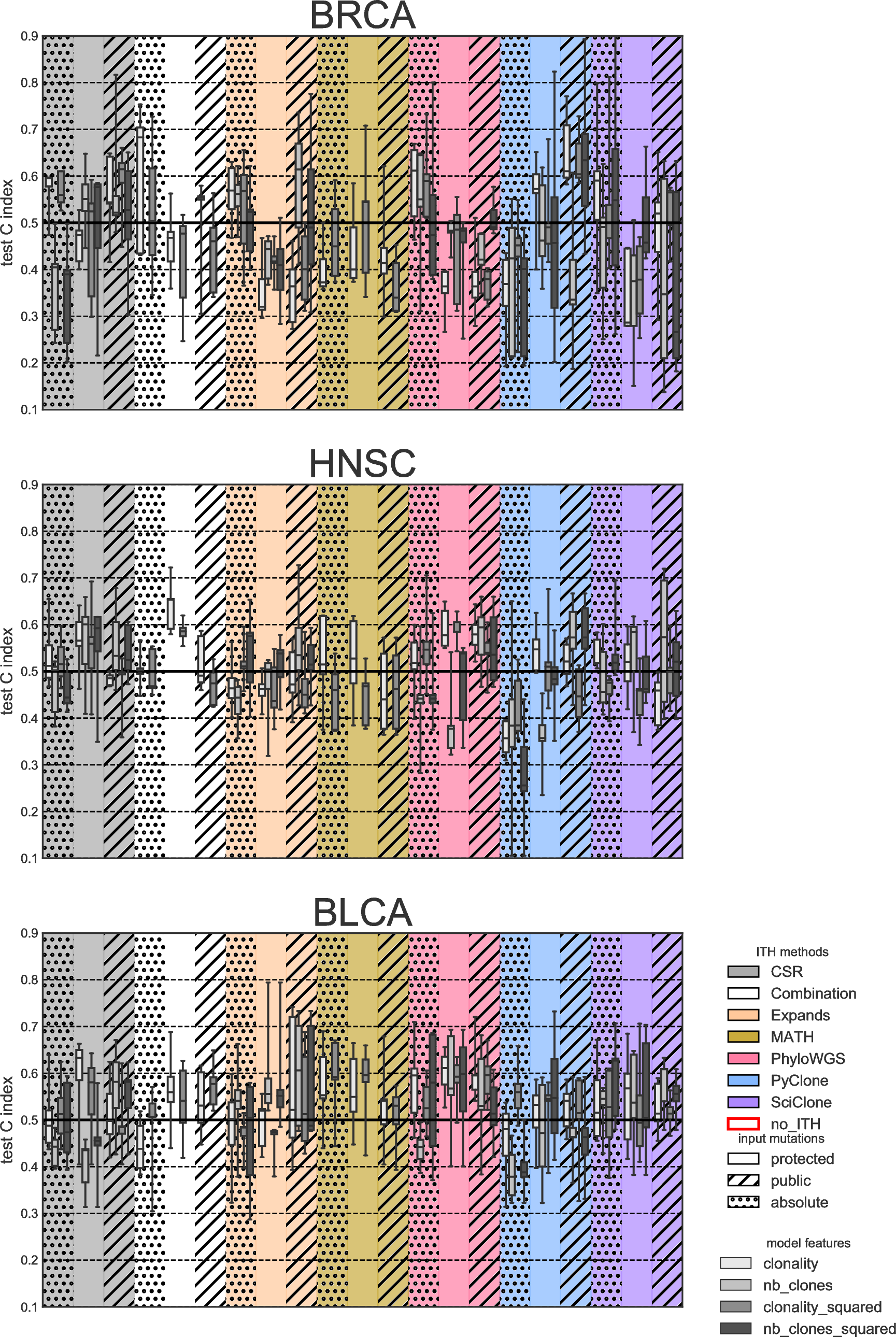

Supplement: S1 Fig — In each plot, the background color indicates the ITH method used. Each method is tested on protected or public mutations (hashed). For each method, we assess the ability to predict survival with a survival SVM using 4 sets of features: (i) the number of clones alone, (ii) the five custom features which include the number of clones, and (iii) and (iv) the concatenations of features in (i) and (ii) with their squares, to account for possible nonlinear quadratic effects. We observe no clear trend of one of the two sets performs systematically better than the other, and the squared features have not significantly improved results either. (TIF) [file pone.0224143.s001.tif]

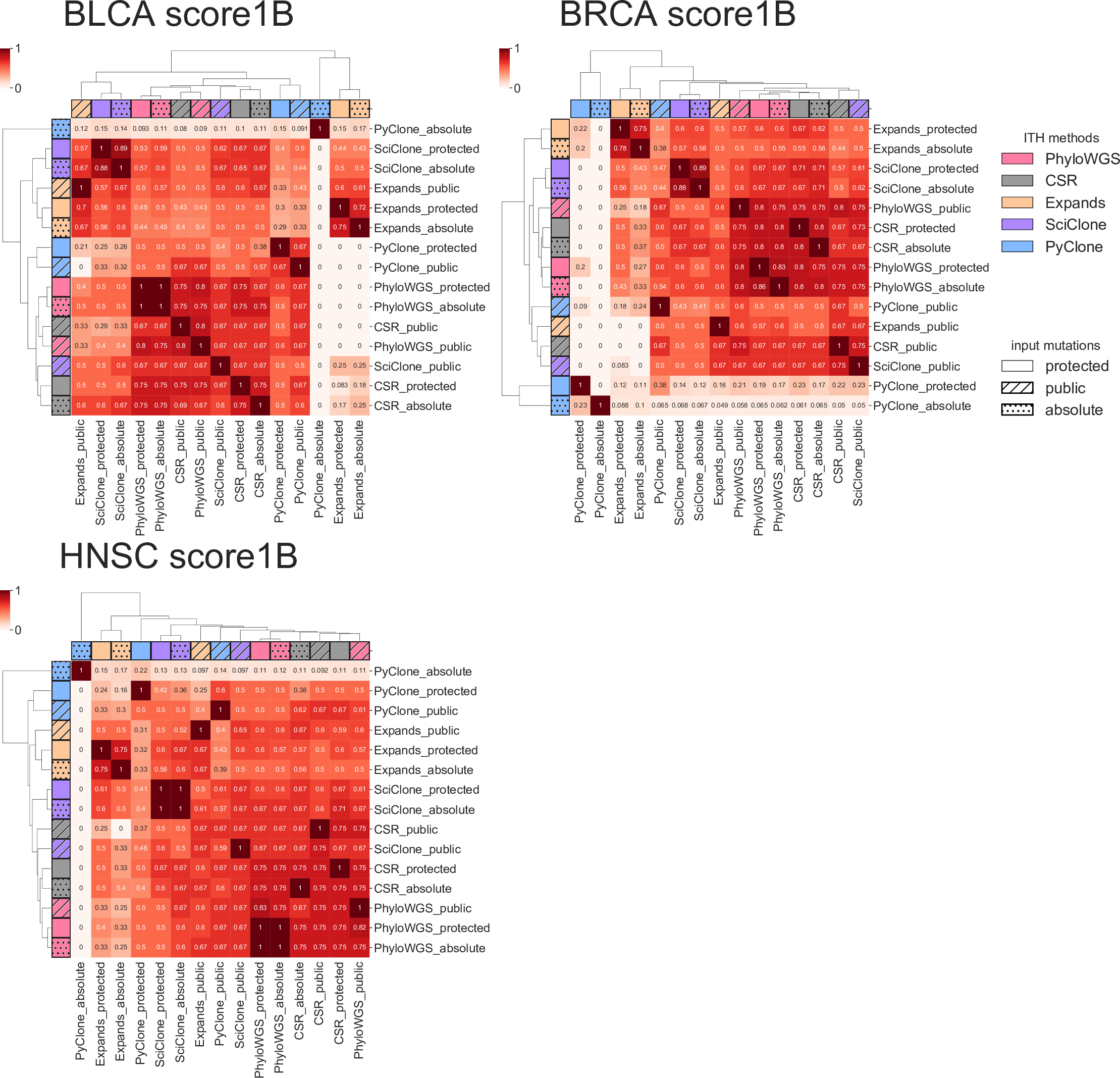

Supplement: S2 Fig — Score1B is a metric designed in [40] penalizes differences between the number of clones inferred in each case in a symmetric way (only the difference matters, either more or fewer clones are detected), following the formula J1+1-min(J1+1,|J2-J1|)J1+1, with J1 and J2 the numbers of clones found by each method. The score was computed for all patients, and this heatmap represents the median score. We observe a particular feature of PyClone, which tends to find a lot (sometimes several dozens) of clones with only one mutation. They were discarded when comparing the number of clones, but not for the computation of metric 1B to ensure consistency with the other metrics. (TIF) [file pone.0224143.s002.tif]

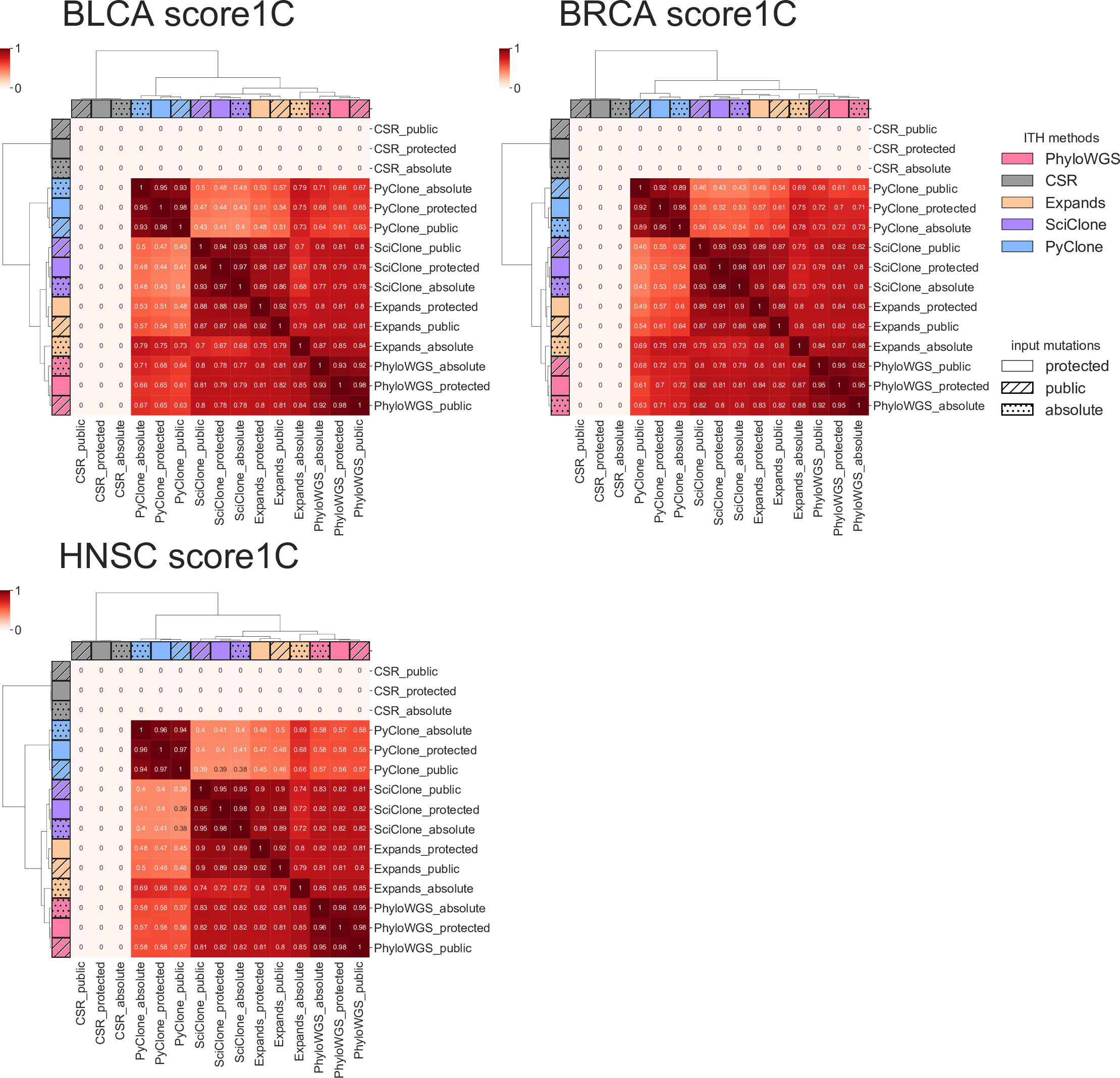

Supplement: S3 Fig — Score1C is a metric designed in [40] that represents the Wasserstein distance between the cancer cell fraction (CCF) distribution resulting from each clone’s mean CCF and number of mutations. Due to the number of single-mutation clones of PyClone, the resulting distribution is quite different from the other cases. As CSR only takes as input the mutation attribution to clones by other methods, without taking into account their CCF, we did not compute score1C for that method. The score was computed for all patients, and this heatmap represents the median score. (TIF) [file pone.0224143.s003.tif]

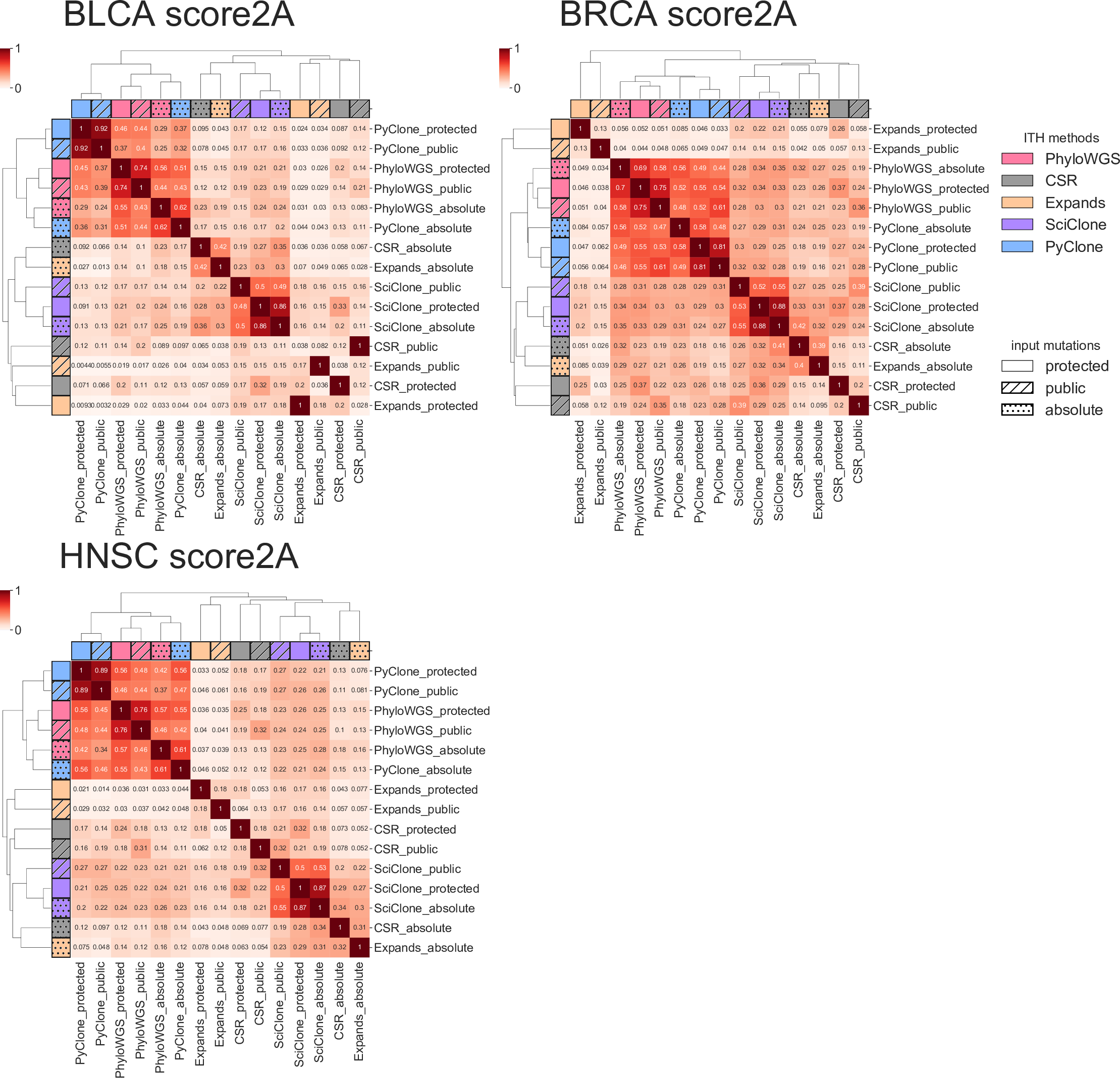

Supplement: S4 Fig — Score2A is a metric designed in [40] that assesses the similarity of the mutation clustering resulting from subclonal reconstruction (see Methods for details). We recover the previously observed pattern that PyClone and PhyloWGS are the closest methods. The score was computed for all patients, and this heatmap represents the median score. (TIF) [file pone.0224143.s004.tif]
