# Supplementary material for: Assessing reliability of intra-tumor heterogeneity estimates from single sample whole exome sequencing data
Source: PLoS One. 2019 Nov 7;14(11):e0224143. doi: 10.1371/journal.pone.0224143 (PMC6837753; doi:10.1371/journal.pone.0224143)
Supplement: S1 Table — Summary statistics of the number of protected and public mutations per sample for BRCA, BLCA and HNSC samples. The protected set corresponds to raw variant calling outputs. The public set corresponds to publicly available SNV calls. (PDF) [file pone.0224143.s005.pdf]

	BRCA	BLCA	HNSC
number of samples	962	351	445
**Protected mutations**			
average	530	735	456
std	1,189	890	544
min	79	61	31
median	342	525	340
max	21,821	12,774	7,941
**Public mutations**			
average	121	352	202
std	375	426	271
min	1	2	1
median	63	241	140
max	7,919	5,478	3,935
