# Supplementary material for: Assessing reliability of intra-tumor heterogeneity estimates from single sample whole exome sequencing data
Source: PLoS One. 2019 Nov 7;14(11):e0224143. doi: 10.1371/journal.pone.0224143 (PMC6837753; doi:10.1371/journal.pone.0224143)
Supplement: S2 Table — Variable significantly associated with survival are shaded. (PDF) [file pone.0224143.s006.pdf]

Variable	Hazard ratio	P-value	Corrected P-value
age_at_diagnosis	1.032023	4.328538e-05	0.000577
PROSPECTIVE_COLLECTION_NO	0.885701	4.431127e-01	0.631390
PROSPECTIVE_COLLECTION_YES	1.129050	4.431127e-01	0.631390
RETROSPECTIVE_COLLECTION_NO	1.138738	4.114412e-01	0.631390
RETROSPECTIVE_COLLECTION_YES	0.885701	4.431127e-01	0.631390
SEX_Female	1.114329	5.131653e-01	0.651638
SEX_Male	0.897401	5.131653e-01	0.651638
RACE_WHITE	1.144930	5.054529e-01	0.651638
HISTORY_OTHER_MALIGNANCY_No	1.100403	5.660149e-01	0.696634
HISTORY_OTHER_MALIGNANCY_Yes	0.908758	5.660149e-01	0.696634
NONINVASIVE_BLADDER_HISTORY_NO	0.878835	3.891088e-01	0.610367
NONINVASIVE_BLADDER_HISTORY_YES	0.868563	4.940541e-01	0.651638
NONINVASIVE_BLADDER_HISTORY_[Not Available]	1.298013	1.113381e-01	0.329891
NONINVASIVE_BLADDER_CA_TX_TYPE_[Not Applicable]	0.878835	3.891088e-01	0.610367
NONINVASIVE_BLADDER_CA_TX_TYPE_[Not Available]	1.225435	2.146545e-01	0.490639
TX_90DAYS_POST_RESECTION_[Not Applicable]	0.878835	3.891088e-01	0.610367
TX_90DAYS_POST_RESECTION_[Not Available]	1.329106	6.724015e-02	0.244510
TX_COMPLETE_RESPONSE_[Not Applicable]	0.878835	3.891088e-01	0.610367
TX_COMPLETE_RESPONSE_[Not Available]	1.183784	2.774378e-01	0.539748
TX_INDUCTION_COURSES_INDICATOR_[Not Applicable]	0.878835	3.891088e-01	0.610367
TX_INDUCTION_COURSES_INDICATOR_[Not Available]	1.234697	1.748396e-01	0.436750
TX_MAINTENANCE_COURSES_INDICATOR_[Not Applicable]	0.878835	3.891088e-01	0.610367
TX_MAINTENANCE_COURSES_INDICATOR_[Not Available]	1.233463	1.768826e-01	0.436750
OCCUPATION_CURRENT_Retired	0.917140	6.255270e-01	0.733401
OCCUPATION_CURRENT_[Not Available]	1.271108	1.467738e-01	0.404893
OCCUPATION_CURRENT_retired	0.960885	8.446695e-01	0.889126
OCCUPATION_PRIMARY_[Not Available]	1.295025	9.419336e-02	0.301419
OCCUPATION_PRIMARY_CHEMICAL_EXPOSURE_[Not Avail...	1.765620	4.091600e-03	0.032733
OCCUPATION_PRIMARY_INDUSTRY_[Not Available]	1.160094	3.247413e-01	0.590439
FAMILY_HISTORY_CANCER_RELATIONSHIP_[Not Available]	1.226713	1.856187e-01	0.436750
FAMILY_HISTORY_CANCER_TYPE_[Not Available]	1.226713	1.856187e-01	0.436750
RADIATION_TREATMENT_ADJUVANT_NO	0.712057	2.632089e-02	0.131604
RADIATION_TREATMENT_ADJUVANT_[Not Available]	1.354145	5.230845e-02	0.210985
PHARMACEUTICAL_TX_ADJUVANT_NO	0.986814	9.296012e-01	0.941368
PHARMACEUTICAL_TX_ADJUVANT_YES	0.676359	4.539278e-02	0.201746
PHARMACEUTICAL_TX_ADJUVANT_[Not Available]	1.387004	3.427365e-02	0.161288
HISTOLOGICAL_SUBTYPE_Non-Papillary	1.364821	7.158132e-02	0.248979
HISTOLOGICAL_SUBTYPE_Papillary	0.673148	2.630851e-02	0.131604
METHOD_OF_INITIAL_SAMPLE_PROCUREMENT_Other meth...	0.813722	4.457771e-01	0.631390
METHOD_OF_INITIAL_SAMPLE_PROCUREMENT_Transureth...	1.234871	2.692175e-01	0.539748
METHOD_OF_INITIAL_SAMPLE_PROCUREMENT_OTHER_[Not...	1.290044	3.459972e-01	0.610367
AJCC_STAGING_EDITION_6th	0.931002	6.427039e-01	0.733401
AJCC_STAGING_EDITION_7th	1.061071	7.031650e-01	0.760178
ANGIOLYMPHATIC_INVASION_NO	0.485392	3.934074e-05	0.000577
ANGIOLYMPHATIC_INVASION_YES	1.818002	6.992848e-05	0.000799
ANGIOLYMPHATIC_INVASION_[Not Available]	1.282364	1.626703e-01	0.433787
LYMPH_NODES_EXAMINED_NO	0.861162	4.914651e-01	0.651638
LYMPH_NODES_EXAMINED_YES	1.144075	4.498650e-01	0.631390

EXTRACAPSULAR_EXTENSION_NO	1.101253	5.846915e-01	0.708717
EXTRACAPSULAR_EXTENSION_YES	1.411088	5.538363e-02	0.210985
EXTRACAPSULAR_EXTENSION_[Not Available]	0.748263	5.291460e-02	0.210985
EXTRACAPSULAR_EXTENSION_PRESENT_[Not Available]	0.804289	2.901143e-01	0.539748
METASTATIC_SITE_Lymph node only	1.687527	6.731283e-03	0.041832
METASTATIC_SITE_None	0.641938	5.729977e-03	0.041673
METASTATIC_SITE_[Not Available]	1.050773	7.417814e-01	0.791234
AJCC_PATHOLOGIC_TUMOR_STAGE_Stage II	0.457221	3.329395e-05	0.000577
AJCC_PATHOLOGIC_TUMOR_STAGE_Stage III	0.840677	2.814664e-01	0.539748
AJCC_PATHOLOGIC_TUMOR_STAGE_Stage IV	2.264256	4.728912e-08	0.000004
INCIDENTAL_PROSTATE_CANCER_NO	1.002834	9.852841e-01	0.985284
INCIDENTAL_PROSTATE_CANCER_YES	0.926590	6.685831e-01	0.742870
INCIDENTAL_PROSTATE_CANCER_[Not Available]	1.107850	6.188245e-01	0.733401
AJCC_INCIDENTAL_PROSTATE_CANCER_[Not Available]	1.135748	4.866109e-01	0.651638
PRIMARY_SITE_Bladder - NOS	0.839975	2.465470e-01	0.533075
CLIN_T_STAGE_T2	1.072482	6.867865e-01	0.752643
CLIN_T_STAGE_[Not Available]	0.801825	1.408015e-01	0.402290
ICD_10_C67.2	0.981491	9.276451e-01	0.941368
ICD_10_C67.9	0.934338	6.508937e-01	0.733401
ICD_O_3_HISTOLOGY_8120/3	1.264392	2.765995e-01	0.539748
ICD_O_3_HISTOLOGY_8130/3	0.790498	2.838725e-01	0.539748
ICD_O_3_SITE_C67.2	0.981491	9.276451e-01	0.941368
ICD_O_3_SITE_C67.9	0.934338	6.508937e-01	0.733401
TISSUE_SOURCE_SITE_DK	0.563206	2.178870e-02	0.124507
TISSUE_SOURCE_SITE_XF	1.263603	2.246627e-01	0.499250
AJCC_TUMOR_PATHOLOGIC_PT_simple_T2	0.515628	4.470852e-04	0.004471
AJCC_TUMOR_PATHOLOGIC_PT_simple_T3	1.271201	1.094625e-01	0.329891
AJCC_TUMOR_PATHOLOGIC_PT_simple_T4	1.880901	9.411876e-04	0.008366
AJCC_NODES_PATHOLOGIC_PN_simple_N0	0.450186	1.450583e-07	0.000006
AJCC_NODES_PATHOLOGIC_PN_simple_N2	2.130213	5.222366e-06	0.000139
AJCC_METASTASIS_PATHOLOGIC_PM_simple_M0	0.663211	6.797682e-03	0.041832
AJCC_METASTASIS_PATHOLOGIC_PM_simple_MX	1.305408	7.476644e-02	0.249221
