# Supplementary material for: Assessing reliability of intra-tumor heterogeneity estimates from single sample whole exome sequencing data
Source: PLoS One. 2019 Nov 7;14(11):e0224143. doi: 10.1371/journal.pone.0224143 (PMC6837753; doi:10.1371/journal.pone.0224143)
Supplement: S3 Table — Variable significantly associated with survival are shaded. (PDF) [file pone.0224143.s007.pdf]

Variable	Hazard ratio	P-value	Corrected P-value
age_at_diagnosis	1.032118	4.566898e-07	1.077788e-05
PROSPECTIVE_COLLECTION_NO	0.602422	4.530180e-02	1.243166e-01
PROSPECTIVE_COLLECTION_YES	1.686136	3.888498e-02	1.176520e-01
RETROSPECTIVE_COLLECTION_NO	1.686136	3.888498e-02	1.176520e-01
RETROSPECTIVE_COLLECTION_YES	0.602422	4.530180e-02	1.243166e-01
MENOPAUSE_STATUS_Post (prior bilateral ovariect...	1.324224	9.572180e-02	2.026486e-01
MENOPAUSE_STATUS_Pre (¡6 months since LMP AND n...	0.431889	8.809889e-04	5.887468e-03
MENOPAUSE_STATUS_[Not Available]	2.943808	9.817809e-07	1.930836e-05
RACE_BLACK OR AFRICAN AMERICAN	1.215466	3.390936e-01	5.275821e-01
RACE_WHITE	0.825045	2.987240e-01	4.828689e-01
RACE_[Not Available]	1.274439	5.361448e-01	6.741716e-01
ETHNICITY_NOT HISPANIC OR LATINO	2.207632	1.176302e-02	5.552148e-02
ETHNICITY_[Not Available]	0.603636	1.259999e-01	2.447704e-01
HISTORY_OTHER_MALIGNANCY_No	0.669493	2.469654e-01	4.047489e-01
HISTORY_OTHER_MALIGNANCY_Yes	1.501028	2.411757e-01	4.016651e-01
RADIATION_TREATMENT_ADJUVANT_NO	0.825072	6.454644e-01	7.693415e-01
RADIATION_TREATMENT_ADJUVANT_YES	1.184551	6.240900e-01	7.525332e-01
RADIATION_TREATMENT_ADJUVANT_[Not Available]	0.991267	9.744419e-01	9.936324e-01
PHARMACEUTICAL_TX_ADJUVANT_YES	0.893189	7.194462e-01	8.162947e-01
PHARMACEUTICAL_TX_ADJUVANT_[Not Available]	1.001332	9.961223e-01	9.961223e-01
METHOD_OF_INITIAL_SAMPLE_PROCUREMENT_Core needl...	0.545734	3.371119e-04	2.841372e-03
METHOD_OF_INITIAL_SAMPLE_PROCUREMENT_Fine needl...	1.669895	1.358907e-02	5.938927e-02
METHOD_OF_INITIAL_SAMPLE_PROCUREMENT_Other meth...	1.009070	9.765300e-01	9.936324e-01
METHOD_OF_INITIAL_SAMPLE_PROCUREMENT_Tumor rese...	0.740284	3.397987e-01	5.275821e-01
METHOD_OF_INITIAL_SAMPLE_PROCUREMENT_[Not Avail...	1.944929	3.630026e-02	1.157684e-01
METHOD_OF_INITIAL_SAMPLE_PROCUREMENT_OTHER_[Not...	0.994946	9.868232e-01	9.952576e-01
SURGICAL_PROCEDURE_FIRST_Lumpectomy	0.690083	9.704508e-02	2.026486e-01
SURGICAL_PROCEDURE_FIRST_Modified Radical Maste...	1.476196	2.170723e-02	8.004541e-02
SURGICAL_PROCEDURE_FIRST_Other	0.882243	5.027363e-01	6.448140e-01
SURGICAL_PROCEDURE_FIRST_Simple Mastectomy	0.657380	9.788958e-02	2.026486e-01
SURGICAL_PROCEDURE_FIRST_[Not Available]	2.480055	2.729308e-03	1.463901e-02
FIRST_SURGICAL_PROCEDURE_OTHER_Surgical Resection	1.534372	3.107861e-01	4.955778e-01
FIRST_SURGICAL_PROCEDURE_OTHER_[Not Available]	1.084261	6.601793e-01	7.790115e-01
PATH_MARGIN_Negative	0.328745	2.487080e-11	1.467377e-09
PATH_MARGIN_Positive	1.456750	1.265338e-01	2.447704e-01
PATH_MARGIN_[Not Available]	4.295169	1.280242e-14	1.510685e-12
SURGERY_FOR_POSITIVE_MARGINS_[Not Available]	0.881873	7.014933e-01	8.141488e-01
MARGIN_STATUS_REEXCISION_Negative	1.189978	5.498957e-01	6.830283e-01
MARGIN_STATUS_REEXCISION_[Not Available]	0.788031	3.970790e-01	5.714063e-01
STAGING_SYSTEM_Axillary lymph node dissection a...	1.147628	4.478192e-01	6.047185e-01
STAGING_SYSTEM_Sentinel lymph node biopsy plus ...	0.471890	9.803200e-04	6.088303e-03
STAGING_SYSTEM_Sentinel node biopsy alone	0.517762	9.212199e-03	4.529331e-02
STAGING_SYSTEM_[Not Available]	2.306054	2.789049e-06	3.502342e-05
MICROMET_DETECTION_BY_IHC_NO	0.983191	9.205373e-01	9.860025e-01
MICROMET_DETECTION_BY_IHC_YES	0.330809	1.172534e-05	1.257809e-04
MICROMET_DETECTION_BY_IHC_[Not Available]	2.187550	2.968086e-06	3.502342e-05
LYMPH_NODES_EXAMINED_YES	1.127012	4.842736e-01	6.279592e-01

LYMPH_NODES_EXAMINED_[Not Available]	0.789955	1.767459e-01	3.160002e-01
AJCC_STAGING_EDITION_5th	2.522972	2.425021e-06	3.502342e-05
AJCC_STAGING_EDITION_6th	0.391254	3.279076e-07	9.673274e-06
AJCC_STAGING_EDITION_7th	1.553473	5.971021e-02	1.455199e-01
AJCC_STAGING_EDITION_[Not Available]	0.905993	7.048542e-01	8.141488e-01
AJCC_PATHOLOGIC_TUMOR_STAGE_Stage I	0.623674	1.050290e-01	2.136796e-01
AJCC_PATHOLOGIC_TUMOR_STAGE_Stage IA	0.246046	1.650590e-02	6.956059e-02
AJCC_PATHOLOGIC_TUMOR_STAGE_Stage IIA	0.697084	6.472348e-02	1.510602e-01
AJCC_PATHOLOGIC_TUMOR_STAGE_Stage IIB	0.828285	3.501038e-01	5.365227e-01
AJCC_PATHOLOGIC_TUMOR_STAGE_Stage IIIA	1.293391	2.416799e-01	4.016651e-01
AJCC_PATHOLOGIC_TUMOR_STAGE_Stage IIIC	1.898479	6.528872e-02	1.510602e-01
ER_STATUS_BY_IHC_Negative	1.272320	1.906432e-01	3.308221e-01
ER_STATUS_BY_IHC_Positive	0.662619	1.727163e-02	7.027765e-02
ER_STATUS_IHC_PERCENT_POSITIVE_90-99%	0.370064	2.554685e-03	1.435490e-02
ER_STATUS_IHC_PERCENT_POSITIVE_¡10%	0.771901	4.520339e-01	6.047185e-01
ER_STATUS_IHC_PERCENT_POSITIVE_[Not Available]	2.276830	1.936838e-05	1.758053e-04
ER_POSITIVITY_SCALE_USED_3 Point Scale	0.369684	5.031369e-02	1.349322e-01
ER_POSITIVITY_SCALE_USED_[Not Available]	2.435285	3.311614e-02	1.116487e-01
ER_POSITIVITY_SCALE_OTHER_[Not Available]	1.595260	1.827359e-01	3.218334e-01
BRACHYTHERAPY_TOTAL_DOSE_POINT_A_[Not Available]	0.994099	9.767911e-01	9.936324e-01
PR_STATUS_BY_IHC_Negative	1.257745	1.746637e-01	3.160002e-01
PR_STATUS_BY_IHC_Positive	0.717066	4.336782e-02	1.243166e-01
PR_STATUS_IHC_PERCENT_POSITIVE_90-99%	0.082244	1.281943e-02	5.818049e-02
PR_STATUS_IHC_PERCENT_POSITIVE_¡10%	0.987074	9.609962e-01	9.936324e-01
PR_STATUS_IHC_PERCENT_POSITIVE_[Not Available]	2.440652	1.832734e-05	1.758053e-04
PR_POSITIVITY_SCALE_USED_3 Point Scale	0.401865	7.305251e-02	1.626452e-01
PR_POSITIVITY_SCALE_USED_[Not Available]	2.235079	5.428402e-02	1.411211e-01
PR_POSITIVITY_IHC_INTENSITY_SCORE_3+	0.127355	4.012324e-02	1.183636e-01
PR_POSITIVITY_IHC_INTENSITY_SCORE_[Not Available]	3.001282	2.553857e-03	1.435490e-02
PR_POSITIVITY_SCALE_OTHER_[Not Available]	1.327000	3.966578e-01	5.714063e-01
PR_POSITIVITY_DEFINE_METHOD_[Not Available]	1.008404	9.676018e-01	9.936324e-01
IHC_HER2_Equivocal	0.601537	5.547120e-02	1.411211e-01
IHC_HER2_Negative	0.791443	1.711526e-01	3.155626e-01
IHC_HER2_Positive	1.504160	7.472326e-02	1.632842e-01
IHC_HER2_[Not Available]	1.460324	3.585853e-02	1.157684e-01
HER2_IHC_PERCENT_POSITIVE_¡10%	0.408510	2.110215e-02	8.004541e-02
HER2_IHC_PERCENT_POSITIVE_[Not Available]	1.864321	2.281919e-02	8.159588e-02
HER2_POSITIVITY_METHOD_TEXT_[Not Available]	2.593860	6.042777e-02	1.455199e-01
HER2_FISH_STATUS_Negative	0.739080	1.150908e-01	2.301816e-01
HER2_FISH_STATUS_Positive	0.880007	7.106553e-01	8.141488e-01
HER2_FISH_STATUS_[Not Available]	1.385428	6.801151e-02	1.543338e-01
HER2_COPY_NUMBER_[Not Available]	1.645350	1.487810e-01	2.831638e-01
CENT17_COPY_NUMBER_[Not Available]	1.548415	2.047798e-01	3.502032e-01
PRIMARY_SITE_Left	1.945749	7.591896e-04	5.599023e-03
PRIMARY_SITE_Left Upper Inner Quadrant	0.734898	4.268913e-01	6.047185e-01
PRIMARY_SITE_Left Upper Outer Quadrant	0.826630	4.397503e-01	6.047185e-01
PRIMARY_SITE_Right	0.892605	6.249852e-01	7.525332e-01
PRIMARY_SITE_Right Upper Outer Quadrant	1.138461	6.005181e-01	7.381368e-01
HISTOLOGICAL_DIAGNOSIS_Infiltrating Ductal Carc...	0.969817	8.637923e-01	9.351146e-01
HISTOLOGICAL_DIAGNOSIS_Infiltrating Lobular Car...	0.823077	3.912033e-01	5.714063e-01
ICD_O_3_HISTOLOGY_8500/3	0.949261	7.692879e-01	8.563771e-01
ICD_O_3_HISTOLOGY_8520/3	0.871304	5.370519e-01	6.741716e-01
METASTATIC_TUMOR_INDICATOR_NO	0.641384	2.830420e-02	9.823221e-02
METASTATIC_TUMOR_INDICATOR_[Not Available]	1.149966	4.502190e-01	6.047185e-01

TISSUE_SOURCE_SITE_A2	0.816065	4.561012e-01	6.047185e-01
TISSUE_SOURCE_SITE_A8	1.386201	4.396658e-01	6.047185e-01
TISSUE_SOURCE_SITE_AR	0.426279	7.036257e-03	3.609906e-02
TISSUE_SOURCE_SITE_B6	1.053316	8.202897e-01	9.046185e-01
TISSUE_SOURCE_SITE_BH	2.772661	1.247824e-08	4.908109e-07
TISSUE_SOURCE_SITE_D8	0.947428	9.275108e-01	9.860025e-01
TISSUE_SOURCE_SITE_E2	0.555754	1.612908e-01	3.021003e-01
TISSUE_SOURCE_SITE_E9	1.350339	4.798622e-01	6.279592e-01
AJCC_TUMOR_PATHOLOGIC_PT_simple_T1	0.686002	5.620924e-02	1.411211e-01
AJCC_TUMOR_PATHOLOGIC_PT_simple_T2	0.951778	7.620026e-01	8.563458e-01
AJCC_TUMOR_PATHOLOGIC_PT_simple_T3	1.220149	3.652398e-01	5.525423e-01
AJCC_NODES_PATHOLOGIC_PN_simple_N0	0.428539	2.290188e-06	3.502342e-05
AJCC_NODES_PATHOLOGIC_PN_simple_N1	1.159871	3.741624e-01	5.588754e-01
AJCC_NODES_PATHOLOGIC_PN_simple_N2	1.714021	1.986213e-02	7.812439e-02
AJCC_NODES_PATHOLOGIC_PN_simple_N3	2.564601	6.195610e-04	4.873880e-03
AJCC_METASTASIS_PATHOLOGIC_PM_simple_M0	0.509375	8.980883e-04	5.887468e-03
AJCC_METASTASIS_PATHOLOGIC_PM_simple_MX	1.062431	8.307274e-01	9.076466e-01
