# Supplementary material for: Assessing reliability of intra-tumor heterogeneity estimates from single sample whole exome sequencing data
Source: PLoS One. 2019 Nov 7;14(11):e0224143. doi: 10.1371/journal.pone.0224143 (PMC6837753; doi:10.1371/journal.pone.0224143)
Supplement: S4 Table — Variable significantly associated with survival are shaded. (PDF) [file pone.0224143.s008.pdf]

Variable	Hazard ratio	P-value	Corrected P-value
INITIAL_PATHOLOGIC_DX_YEAR	0.944473	0.000002	0.000085
age_at_diagnosis	1.019742	0.001594	0.012355
PRIMARY_SITE_Floor of mouth	1.633189	0.009866	0.048599
PRIMARY_SITE_Larynx	0.870495	0.392949	0.589424
PRIMARY_SITE_Oral Cavity	1.216594	0.257959	0.436185
PRIMARY_SITE_Oral Tongue	1.027363	0.864976	0.935381
LATERALITY_Left	0.997223	0.985683	0.985683
LATERALITY_Right	0.596169	0.001229	0.010391
LATERALITY_[Not Available]	1.487693	0.003515	0.021791
PROSPECTIVE_COLLECTION_NO	1.275968	0.136430	0.309463
PROSPECTIVE_COLLECTION_YES	0.796014	0.163125	0.337124
RETROSPECTIVE_COLLECTION_NO	0.796014	0.163125	0.337124
RETROSPECTIVE_COLLECTION_YES	1.275968	0.136430	0.309463
SEX_Female	1.382324	0.024394	0.087255
SEX_Male	0.723420	0.024394	0.087255
RACE_WHITE	0.742605	0.111804	0.273433
ETHNICITY_NOT HISPANIC OR LATINO	0.853619	0.414453	0.611812
LYMPH_NODE_NECK_DISSECTION_INDICATOR_NO	1.236683	0.203896	0.403454
LYMPH_NODE_NECK_DISSECTION_INDICATOR_YES	0.814286	0.215391	0.412907
LYMPH_NODE_DISSECTION_METHOD_Functional (Limite...	1.086460	0.540168	0.749786
LYMPH_NODE_DISSECTION_METHOD_Modified Radical N...	0.741422	0.093637	0.241895
LYMPH_NODE_DISSECTION_METHOD_[Not Available]	1.090921	0.583249	0.763974
LYMPH_NODES_EXAMINED_NO	1.470161	0.061855	0.179766
LYMPH_NODES_EXAMINED_YES	0.837805	0.290092	0.481761
PATH_MARGIN_Negative	0.777605	0.079280	0.216854
PATH_MARGIN_Positive	1.727697	0.002205	0.014645
PATH_MARGIN_[Not Available]	0.699698	0.158253	0.337124
AJCC_STAGING_EDITION_6th	1.010909	0.944167	0.955798
AJCC_STAGING_EDITION_7th	0.973421	0.855035	0.935381
AJCC_PATHOLOGIC_TUMOR_STAGE_Stage II	0.668524	0.054444	0.168777
AJCC_PATHOLOGIC_TUMOR_STAGE_Stage III	0.726051	0.114665	0.273433
AJCC_PATHOLOGIC_TUMOR_STAGE_Stage IVA	1.532143	0.001807	0.012924
AJCC_PATHOLOGIC_TUMOR_STAGE_[Not Available]	0.962185	0.849093	0.935381
EXTRACAPSULAR_SPREAD_PATHOLOGIC_Microscopic Ext...	2.254494	0.000001	0.000085
EXTRACAPSULAR_SPREAD_PATHOLOGIC_No Extranodal E...	0.548915	0.000017	0.000392
EXTRACAPSULAR_SPREAD_PATHOLOGIC_[Not Available]	0.990168	0.945521	0.955798
GRADE_G1	0.657592	0.059833	0.179498
GRADE_G2	1.431246	0.010999	0.050448
GRADE_G3	0.957962	0.783689	0.915850
ANGIOLYMPHATIC_INVASION_NO	0.693371	0.009929	0.048599
ANGIOLYMPHATIC_INVASION_YES	1.484568	0.008174	0.047509
ANGIOLYMPHATIC_INVASION_[Not Available]	1.057948	0.692475	0.847371
PERINEURAL_INVASION_NO	0.516015	0.000030	0.000565
PERINEURAL_INVASION_YES	1.588730	0.000670	0.006230
PERINEURAL_INVASION_[Not Available]	1.147166	0.339620	0.535334
HPV_STATUS_P16_Negative	0.856744	0.477818	0.673289
HPV_STATUS_P16_[Not Available]	1.469555	0.052450	0.168202
HPV_STATUS_ISH_Negative	0.842503	0.448772	0.642089
HPV_STATUS_ISH_[Not Available]	1.564703	0.043660	0.145013
TOBACCO_SMOKING_HISTORY_INDICATOR_1	0.853226	0.347479	0.538592

TOBACCO_SMOKING_HISTORY_INDICATOR_2	1.349280	0.033815	0.116475
TOBACCO_SMOKING_HISTORY_INDICATOR_3	0.708845	0.088463	0.235059
TOBACCO_SMOKING_HISTORY_INDICATOR_4	0.949831	0.733565	0.874635
ALCOHOL_HISTORY_DOCUMENTED_NO	1.064729	0.660954	0.841584
ALCOHOL_HISTORY_DOCUMENTED_YES	0.941649	0.669647	0.841584
RADIATION_TREATMENT_ADJUVANT_NO	1.062557	0.787828	0.915850
RADIATION_TREATMENT_ADJUVANT_YES	0.905136	0.549419	0.751412
RADIATION_TREATMENT_ADJUVANT_[Not Available]	1.055628	0.712535	0.860594
PHARMACEUTICAL_TX_ADJUVANT_NO	0.774111	0.144766	0.320553
PHARMACEUTICAL_TX_ADJUVANT_YES	1.454739	0.067970	0.191551
PHARMACEUTICAL_TX_ADJUVANT_[Not Available]	1.032903	0.825634	0.935381
CLIN_N_STAGE_N0	0.853501	0.240473	0.436185
CLIN_N_STAGE_N1	0.915126	0.621348	0.802575
CLIN_N_STAGE_N2b	0.980360	0.916449	0.955798
CLIN_T_STAGE_T2	0.838379	0.251047	0.436185
CLIN_T_STAGE_T3	1.269859	0.104375	0.262348
CLIN_T_STAGE_T4a	1.014869	0.918479	0.955798
CLINICAL_STAGE_Stage II	0.872315	0.435405	0.632698
CLINICAL_STAGE_Stage III	0.978740	0.898501	0.955798
CLINICAL_STAGE_Stage IVA	1.138939	0.338664	0.535334
ICD_10_C02.9	0.970829	0.852993	0.935381
ICD_10_C04.9	2.068995	0.000137	0.001823
ICD_10_C14.8	1.218218	0.254981	0.436185
ICD_10_C32.9	0.914839	0.578172	0.763974
ICD_O_3_HISTOLOGY_8070/3	1.344115	0.221993	0.412907
ICD_O_3_HISTOLOGY_8071/3	0.898413	0.682330	0.846089
ICD_O_3_SITE_C02.9	0.970829	0.852993	0.935381
ICD_O_3_SITE_C04.9	2.068995	0.000137	0.001823
ICD_O_3_SITE_C14.8	1.218218	0.254981	0.436185
ICD_O_3_SITE_C32.9	0.914839	0.578172	0.763974
TISSUE_SOURCE_SITE_CN	1.213414	0.297016	0.484604
TISSUE_SOURCE_SITE_CR	0.431810	0.009643	0.048599
TISSUE_SOURCE_SITE_CV	1.663811	0.000385	0.003978
AJCC_TUMOR_PATHOLOGIC_PT_simple_T2	0.659617	0.011391	0.050448
AJCC_TUMOR_PATHOLOGIC_PT_simple_T3	1.459494	0.016649	0.070379
AJCC_TUMOR_PATHOLOGIC_PT_simple_T4	1.388501	0.017779	0.071889
AJCC_NODES_PATHOLOGIC_PN_simple_N0	0.556349	0.000185	0.002146
AJCC_NODES_PATHOLOGIC_PN_simple_N1	0.576861	0.022426	0.086899
AJCC_NODES_PATHOLOGIC_PN_simple_N2	1.834907	0.000009	0.000292
AJCC_NODES_PATHOLOGIC_PN_simple_NX	1.165686	0.381317	0.581352
AJCC_METASTASIS_PATHOLOGIC_PM_simple_M0	0.818485	0.176511	0.356858
AJCC_METASTASIS_PATHOLOGIC_PM_simple_MX	0.979428	0.928250	0.955798
AJCC_METASTASIS_PATHOLOGIC_PM_simple_[N	1.185902	0.220529	0.412907
