# Supplementary material for: Assessing reliability of intra-tumor heterogeneity estimates from single sample whole exome sequencing data
Source: PLoS One. 2019 Nov 7;14(11):e0224143. doi: 10.1371/journal.pone.0224143 (PMC6837753; doi:10.1371/journal.pone.0224143)
Supplement: S5 Table — Genes Id were matched from tables available at https://github.com/judithabk6/ITH_TCGA/tree/master/external_data. (PDF) [file pone.0224143.s009.pdf]

CellType	Symbol	AffymetrixID	EntrezGene	Gene Ensembl ID
B cells	MS4A1	217418_x_at	931	ENSG00000156738
B cells	TCL1A	209995_s_at	8115	ENSG00000100721
B cells	MS4A1	210356_x_at	931	ENSG00000156738
B cells	TCL1A	39318_at	8115	ENSG00000100721
B cells	HLA-DOB	205671_s_at	3112	ENSG00000241106
B cells	HLA-DOB	205671_s_at	3112	ENSG00000239457
B cells	HLA-DOB	205671_s_at	3112	ENSG00000243496
B cells	HLA-DOB	205671_s_at	3112	ENSG00000241386
B cells	HLA-DOB	205671_s_at	3112	ENSG00000241910
B cells	HLA-DOB	205671_s_at	3112	ENSG00000243612
B cells	PNOC	205901_at	5368	ENSG00000168081
B cells	KIAA0125	206478_at	9834	ENSG00000277059
B cells	KIAA0125	206478_at	9834	ENSG00000226777
B cells	CD19	206398_s_at	930	ENSG00000177455
B cells	CR2	205544_s_at	1380	ENSG00000117322
B cells	IGHG1	213674_x_at	3500	NaN
B cells	FCRL2	221239_s_at	79368	ENSG00000132704
B cells	BLK	206255_at	640	ENSG00000136573
B cells	IGHG1	222285_at	3500	NaN
B cells	COCH	205229_s_at	1690	ENSG00000100473
B cells	OSBPL10	219073_s_at	114884	ENSG00000144645
B cells	IGHA1	215118_s_at	3493	NaN
B cells	TNFRSF17	206641_at	608	ENSG00000048462
B cells	ABCB4	207819_s_at	5244	ENSG00000005471
B cells	BLNK	207655_s_at	29760	ENSG00000095585
B cells	GLDC	204836_at	2731	ENSG00000178445
B cells	MEF2C	209200_at	4208	ENSG00000081189
B cells	MEF2C	209199_s_at	4208	ENSG00000081189
B cells	IGHM	209374_s_at	3507	NaN
B cells	FAM30A	220377_at	29064	NaN
B cells	SPIB	205861_at	6689	ENSG00000269404
B cells	BCL11A	219497_s_at	53335	ENSG00000119866
B cells	GNG7	206896_s_at	2788	ENSG00000176533
B cells	IGKC	215217_at	3514	NaN
B cells	CD72	215925_s_at	971	ENSG00000137101
B cells	MICAL3	212715_s_at	57553	ENSG00000243156
B cells	BCL11A	210347_s_at	53335	ENSG00000119866
B cells	BACH2	221234_s_at	60468	ENSG00000112182
B cells	IGL@	217138_x_at	3535	NaN
B cells	CCR9	207445_s_at	10803	ENSG00000173585
B cells	QRSL1	218948_at	55278	ENSG00000130348
B cells	DTNB	215565_at	1838	ENSG00000138101
B cells	HLA-DQA1	212671_s_at	3117	ENSG00000236418
B cells	HLA-DQA1	212671_s_at	3117	ENSG00000232062
B cells	HLA-DQA1	212671_s_at	3117	ENSG00000223793
B cells	HLA-DQA1	212671_s_at	3117	ENSG00000231526
B cells	HLA-DQA1	212671_s_at	3117	ENSG00000257473
B cells	HLA-DQA1	212671_s_at	3117	ENSG00000228284
B cells	HLA-DQA1	212671_s_at	3117	ENSG00000225890
B cells	HLA-DQA1	212671_s_at	3117	ENSG00000231823
B cells	HLA-DQA1	212671_s_at	3117	ENSG00000206301
B cells	HLA-DQA1	212671_s_at	3117	ENSG00000206305

B cells	HLA-DQA1	212671_s_at	3117	ENSG00000225103
B cells	HLA-DQA1	212671_s_at	3117	ENSG00000233192
B cells	HLA-DQA1	212671_s_at	3117	ENSG00000196735
B cells	HLA-DQA1	212671_s_at	3117	ENSG00000237541
B cells	SCN3A	210432_s_at	6328	ENSG00000153253
B cells	QRSL1	218949_s_at	55278	ENSG00000130348
B cells	SLC15A2	205316_at	6565	ENSG00000163406
T cells	PRKCQ	210038_at	5588	ENSG00000065675
T cells	CD3D	213539_at	915	ENSG00000167286
T cells	CD3G	206804_at	917	ENSG00000160654
T cells	CD28	206545_at	940	ENSG00000178562
T cells	LCK	204891_s_at	3932	ENSG00000182866
T cells	TRAT1	217147_s_at	50852	ENSG00000163519
T cells	PRKCQ	210039_s_at	5588	ENSG00000065675
T cells	BCL11B	219528_s_at	64919	ENSG00000127152
T cells	CD2	205831_at	914	ENSG00000116824
T cells	LCK	204890_s_at	3932	ENSG00000182866
T cells	TRBC1	213193_x_at	28639	NaN
T cells	TRBC1	210915_x_at	28639	NaN
T cells	TRA@	209670_at	28755	NaN
T cells	ITM2A	202747_s_at	9452	ENSG00000078596
T cells	SH2D1A	210116_at	4068	ENSG00000183918
T cells	CD6	213958_at	923	ENSG00000013725
T cells	CD96	206761_at	10225	ENSG00000153283
T cells	NCALD	211685_s_at	83988	ENSG00000104490
T cells	GIMAP5	218805_at	55340	ENSG00000196329
T cells	TRA@	209671_x_at	6955	NaN
T cells	CD3E	205456_at	916	ENSG00000198851
T cells	SKAP1	205790_at	8631	ENSG00000141293
T cells	TRA@	213830_at	6955	NaN
T cells	TRA@	216191_s_at	6955	NaN
T helper cells	ICOS	210439_at	29851	ENSG00000163600
T helper cells	LRBA	214109_at	987	ENSG00000198589
T helper cells	ITM2A	202746_at	9452	ENSG00000078596
T helper cells	FAM111A	218248_at	63901	ENSG00000166801
T helper cells	PHF10	219126_at	55274	ENSG00000130024
T helper cells	NUP107	218768_at	57122	ENSG00000111581
T helper cells	SEC24C	202361_at	9632	ENSG00000176986
T helper cells	NAP1L4	201414_s_at	4676	ENSG00000205531
T helper cells	NAP1L4	201414_s_at	4676	ENSG00000273562
T helper cells	BATF	205965_at	10538	ENSG00000156127
T helper cells	ASF1A	203428_s_at	25842	ENSG00000111875
T helper cells	FRYL	212546_s_at	285527	ENSG00000075539
T helper cells	FUSIP1	213594_x_at	10772	ENSG00000188529
T helper cells	TRA@	215524_x_at	10730	ENSG00000136758
T helper cells	TRA@	217412_at	6955	NaN
T helper cells	RPA1	201528_at	6117	ENSG00000132383
T helper cells	UBE2L3	200683_s_at	7332	ENSG00000185651
T helper cells	ANP32B	201306_s_at	10541	ENSG00000136938
T helper cells	DDX50	221699_s_at	79009	ENSG00000107625
T helper cells	C13orf34	219544_at	79866	ENSG00000136122
T helper cells	PPP2R5C	213305_s_at	5527	ENSG00000078304
T helper cells	SLC25A12	203340_s_at	8604	ENSG00000115840
T helper cells	ATF2	205446_s_at	1386	ENSG00000115966

T helper cells	CD28	211856_x_at	940	ENSG00000178562
T helper cells	GOLGA8A	208798_x_at	23015	ENSG00000175265
Tcm	CDC14A	210441_at	8556	ENSG00000079335
Tcm	ATM	208442_s_at	472	ENSG00000149311
Tcm	USP9Y	206624_at	8287	ENSG00000114374
Tcm	PCNX	215175_at	22990	ENSG00000100731
Tcm	ATM	210858_x_at	472	ENSG00000149311
Tcm	FOXP1	215221_at	27086	ENSG00000114861
Tcm	KLF12	206965_at	11278	ENSG00000118922
Tcm	ST3GAL1	215874_at	6482	ENSG00000008513
Tcm	INPP4B	215864_at	8821	ENSG00000109452
Tcm	CASP8	207686_s_at	841	ENSG00000064012
Tcm	MLL	216624_s_at	4297	ENSG00000118058
Tcm	PCM1	209997_x_at	5108	ENSG00000078674
Tcm	RP11-74E24.2	205787_x_at	441155	ENSG00000215817
Tcm	PHC3	215521_at	80012	ENSG00000173889
Tcm	NFATC3	210556_at	4775	ENSG00000072736
Tcm	LOC202134	215133_s_at	202134	ENSG00000182230
Tcm	TIMM8A	210800_at	1678	ENSG00000126953
Tcm	ATF7IP	216197_at	55729	ENSG00000171681
Tcm	REPS1	215201_at	85021	ENSG00000135597
Tcm	PSPC1	215083_at	55269	ENSG00000121390
Tcm	RPP38	215743_at	9397	ENSG00000152465
Tcm	HNRPH1	213472_at	3187	ENSG00000169045
Tcm	STX16	221638_s_at	8675	ENSG00000124222
Tcm	CYLD	214272_at	1540	ENSG00000083799
Tcm	SNRPN	216850_at	6638	ENSG00000128739
Tcm	TRAF3IP3	215275_at	80342	ENSG00000009790
Tcm	NEFL	221805_at	4747	ENSG00000277586
Tcm	POLR2J2	217610_at	246721	ENSG00000228049
Tcm	AQP3	203747_at	360	ENSG00000165272
Tcm	CG030	215105_at	116828	ENSG00000281026
Tcm	PDXDC2	215920_s_at	283970	ENSG00000196696
Tcm	CLUAP1	204576_s_at	23059	ENSG00000103351
Tcm	DOCK9	215041_s_at	23348	ENSG00000088387
Tcm	CYorf15B	214131_at	84663	NaN
Tcm	CREBZF	213584_s_at	58487	ENSG00000137504
Tcm	CEP68	207971_s_at	23177	ENSG00000011523
Tcm	TXK	206828_at	7294	ENSG00000074966
Tcm	SLC7A6	203578_s_at	9057	ENSG00000103064
Tcm	FYB	205285_s_at	2533	ENSG00000082074
Tcm	MAP3K1	214786_at	4214	ENSG00000095015
Tem	TRA@	217397_at	6955	NaN
Tem	PRKY	206279_at	5616	ENSG00000099725
Tem	VIL2	217230_at	7430	ENSG00000092820
Tem	GDPD5	32502_at	81544	ENSG00000158555
Tem	CCR2	206978_at	1231	NaN
Tem	MEFV	208262_x_at	4210	ENSG00000103313
Tem	C7orf54	210109_at	27099	ENSG00000279078
Tem	FLI1	210786_s_at	2313	ENSG00000151702
Tem	TBC1D5	201815_s_at	9779	ENSG00000131374
Tem	DDX17	208719_s_at	10521	ENSG00000100201
Tem	AKT3	212609_s_at	10000	ENSG00000275199
Tem	AKT3	212609_s_at	10000	ENSG00000117020

Tem	EWSR1	211825_s_at	2130	ENSG00000182944
Tem	TBCD	201759_at	6904	ENSG00000278759
Tem	TBCD	201759_at	6904	ENSG00000141556
Tem	CCR2	207794_at	1231	NaN
Tem	NFATC4	205897_at	4776	ENSG00000100968
Tem	LTK	207106_s_at	4058	ENSG00000062524
Th1 cells	IFNG	210354_at	3458	ENSG00000111537
Th1 cells	LTA	206975_at	4049	ENSG00000238130
Th1 cells	LTA	206975_at	4049	ENSG00000173503
Th1 cells	LTA	206975_at	4049	ENSG00000231408
Th1 cells	LTA	206975_at	4049	ENSG00000226979
Th1 cells	LTA	206975_at	4049	ENSG00000230279
Th1 cells	LTA	206975_at	4049	ENSG00000226275
Th1 cells	LTA	206975_at	4049	ENSG00000223919
Th1 cells	APBB2	213419_at	323	ENSG00000163697
Th1 cells	DOK5	214844_s_at	55816	ENSG00000101134
Th1 cells	IL12RB2	206999_at	3595	ENSG00000081985
Th1 cells	APBB2	40148_at	323	ENSG00000163697
Th1 cells	APOD	201525_at	347	ENSG00000189058
Th1 cells	ZBTB32	220118_at	27033	ENSG00000011590
Th1 cells	CD38	205692_s_at	952	ENSG00000004468
Th1 cells	CSF2	210229_s_at	1437	ENSG00000164400
Th1 cells	CTLA4	221331_x_at	1493	ENSG00000163599
Th1 cells	CD70	206508_at	970	ENSG00000125726
Th1 cells	DPP4	211478_s_at	1803	ENSG00000197635
Th1 cells	EGFL6	219454_at	25975	ENSG00000198759
Th1 cells	BST2	201641_at	684	ENSG00000130303
Th1 cells	DUSP5	209457_at	1847	ENSG00000138166
Th1 cells	LRP8	205282_at	7804	ENSG00000157193
Th1 cells	IL22	221165_s_at	50616	ENSG00000127318
Th1 cells	DGKI	206806_at	9162	ENSG00000157680
Th1 cells	CCL4	204103_at	6351	ENSG00000275824
Th1 cells	CCL4	204103_at	6351	ENSG00000275302
Th1 cells	CCL4	204103_at	6351	ENSG00000277943
Th1 cells	DPP4	203716_s_at	1803	ENSG00000197635
Th1 cells	GGT1	211417_x_at	2678	ENSG00000100031
Th1 cells	LRRN3	209840_s_at	54674	ENSG00000173114
Th1 cells	SYNGR3	205691_at	9143	ENSG00000127561
Th1 cells	ATP9A	212062_at	10079	ENSG00000054793
Th1 cells	BTG3	205548_s_at	10950	ENSG00000281484
Th1 cells	BTG3	205548_s_at	10950	ENSG00000154640
Th1 cells	CMAH	210571_s_at	8418	NaN
Th1 cells	HBEGF	38037_at	1839	ENSG00000113070
Th1 cells	SGCB	205120_s_at	6443	ENSG00000163069
Th2 cells	PMCH	206942_s_at	5367	ENSG00000183395
Th2 cells	AHI1	220841_s_at	54806	ENSG00000135541
Th2 cells	PTGIS	208131_s_at	5740	ENSG00000124212
Th2 cells	AHI1	220842_at	54806	ENSG00000135541
Th2 cells	CXCR6	211469_s_at	10663	ENSG00000172215
Th2 cells	EVI5	209717_at	7813	ENSG00000067208
Th2 cells	AHI1	221569_at	54806	ENSG00000135541
Th2 cells	IL26	221111_at	55801	ENSG00000111536
Th2 cells	MB	204179_at	4151	ENSG00000198125
Th2 cells	NEIL3	219502_at	55247	ENSG00000109674

Th2 cells	GSTA4	202967_at	2941	ENSG00000170899
Th2 cells	PHEX	210617_at	5251	ENSG00000102174
Th2 cells	SMAD2	203076_s_at	4087	ENSG00000175387
Th2 cells	CENPF	209172_s_at	1063	ENSG00000117724
Th2 cells	ANK1	208353_x_at	286	ENSG00000029534
Th2 cells	ADCY1	213245_at	107	ENSG00000164742
Th2 cells	AI582773	214373_at	728210	NaN
Th2 cells	LAIR2	207509_s_at	3904	ENSG00000277335
Th2 cells	LAIR2	207509_s_at	3904	ENSG00000274084
Th2 cells	LAIR2	207509_s_at	3904	ENSG00000167618
Th2 cells	LAIR2	207509_s_at	3904	ENSG00000275819
Th2 cells	SNRPD1	202691_at	6632	ENSG00000167088
Th2 cells	CXCR6	206974_at	10663	ENSG00000172215
Th2 cells	MICAL2	212472_at	9645	ENSG00000133816
Th2 cells	DHFR	202534_x_at	1719	ENSG00000228716
Th2 cells	SMAD2	203077_s_at	4087	ENSG00000175387
Th2 cells	WDHD1	204728_s_at	11169	ENSG00000198554
Th2 cells	BIRC5	210334_x_at	332	ENSG00000089685
Th2 cells	DHFR	48808_at	1719	ENSG00000228716
Th2 cells	SLC39A14	212110_at	23516	ENSG00000104635
Th2 cells	HELLS	220085_at	3070	ENSG00000119969
Th2 cells	LIMA1	217892_s_at	51474	ENSG00000050405
Th2 cells	CDC25C	205167_s_at	995	ENSG00000158402
Th2 cells	CDC7	204510_at	8317	ENSG00000097046
Th2 cells	GATA3	209602_s_at	2625	ENSG00000107485
TFH	CHI3L2	213060_s_at	1117	ENSG00000064886
TFH	CXCL13	205242_at	10563	ENSG00000156234
TFH	MYO7A	33197_at	4647	ENSG00000137474
TFH	CHGB	204260_at	1114	ENSG00000089199
TFH	MYO7A	208189_s_at	4647	ENSG00000137474
TFH	ICA1	210547_x_at	3382	ENSG00000003147
TFH	HEY1	218839_at	23462	ENSG00000164683
TFH	CDK5R1	204995_at	8851	ENSG00000176749
TFH	ST8SIA1	210073_at	6489	ENSG00000111728
TFH	PDCD1	207634_at	5133	ENSG00000276977
TFH	PDCD1	207634_at	5133	ENSG00000188389
TFH	BLR1	216734_s_at	643	ENSG00000160683
TFH	KIAA1324	221874_at	57535	ENSG00000116299
TFH	PVALB	205336_at	5816	ENSG00000100362
TFH	PVALB	205336_at	5816	ENSG00000274665
TFH	ICA1	207949_s_at	3382	ENSG00000003147
TFH	TSHR	210055_at	7253	ENSG00000165409
TFH	C18orf1	209574_s_at	753	ENSG00000168675
TFH	HEY1	44783_s_at	23462	ENSG00000164683
TFH	TOX	204529_s_at	9760	ENSG00000198846
TFH	BLR1	206126_at	643	ENSG00000160683
TFH	SLC7A10	220868_s_at	56301	ENSG00000130876
TFH	SMAD1	210993_s_at	4086	ENSG00000170365
TFH	POMT1	218476_at	10585	ENSG00000130714
TFH	PASK	216945_x_at	23178	ENSG00000115687
TFH	MKL2	218259_at	57496	ENSG00000186260
TFH	PTPN13	204201_s_at	5783	ENSG00000163629
TFH	PASK	213534_s_at	23178	ENSG00000115687
TFH	KCNK5	219615_s_at	8645	ENSG00000164626

TFH	C18orf1	207996_s_at	753	ENSG00000168675
TFH	ZNF764	57516_at	92595	ENSG00000169951
TFH	MAF	206363_at	4094	ENSG00000178573
TFH	MYO6	210480_s_at	4646	ENSG00000196586
TFH	SIRPG	220485_s_at	55423	ENSG00000089012
TFH	THADA	54632_at	63892	ENSG00000115970
TFH	THADA	220212_s_at	63892	ENSG00000115970
TFH	MAGEH1	218573_at	28986	ENSG00000187601
TFH	B3GAT1	219521_at	27087	ENSG00000109956
TFH	MAF	209348_s_at	4094	ENSG00000178573
TFH	SH3TC1	219256_s_at	54436	ENSG00000125089
TFH	HIST1H4K	214463_x_at	8362	ENSG00000273542
TFH	STK39	202786_at	27347	ENSG00000198648
Th17 cells	IL17A	208402_at	3605	ENSG00000112115
Th17 cells	IL17A	216876_s_at	3605	ENSG00000112115
Th17 cells	IL17RA	205707_at	23765	ENSG00000177663
Th17 cells	RORC	206419_at	6097	ENSG00000143365
TReg	FOXP3	221333_at	50943	ENSG00000049768
TReg	FOXP3	221334_s_at	50943	ENSG00000049768
CD8 T cells	CD8B	207979_s_at	926	ENSG00000172116
CD8 T cells	CD8A	205758_at	925	ENSG00000153563
CD8 T cells	CD8B	215332_s_at	926	ENSG00000172116
CD8 T cells	PF4	206390_x_at	5196	ENSG00000163737
CD8 T cells	PRR5	47069_at	55615	ENSG00000186654
CD8 T cells	SF1	210172_at	7536	ENSG00000168066
CD8 T cells	LIME1	219541_at	54923	ENSG00000203896
CD8 T cells	DNAJB1	200664_s_at	3337	ENSG00000132002
CD8 T cells	ARHGAP8	219168_s_at	55615	ENSG00000186654
CD8 T cells	GZMM	207460_at	3004	ENSG00000197540
CD8 T cells	SLC16A7	207057_at	9194	ENSG00000118596
CD8 T cells	SFRS7	213649_at	6432	ENSG00000115875
CD8 T cells	APBA2	209871_s_at	321	ENSG00000034053
CD8 T cells	APBA2	209871_s_at	321	ENSG00000276495
CD8 T cells	C4orf15	210054_at	79441	ENSG00000214367
CD8 T cells	LEPROTL1	202595_s_at	23484	ENSG00000104660
CD8 T cells	ZFP36L2	201367_s_at	678	ENSG00000152518
CD8 T cells	GADD45A	203725_at	1647	ENSG00000116717
CD8 T cells	ZFP36L2	201369_s_at	678	ENSG00000152518
CD8 T cells	MYST3	216361_s_at	7994	ENSG00000083168
CD8 T cells	ZEB1	208078_s_at	6935	ENSG00000148516
CD8 T cells	ZNF609	212620_at	23060	ENSG00000180357
CD8 T cells	C12orf47	64432_at	51275	ENSG00000234608
CD8 T cells	THUMPD1	206555_s_at	55623	ENSG00000066654
CD8 T cells	VAMP2	201557_at	6844	ENSG00000220205
CD8 T cells	ZNF91	206059_at	7644	ENSG00000167232
CD8 T cells	ZNF22	218006_s_at	7570	ENSG00000165512
CD8 T cells	TMC6	214958_s_at	11322	ENSG00000141524
CD8 T cells	DNAJB1	200666_s_at	3337	ENSG00000132002
CD8 T cells	FLT3LG	210607_at	2323	ENSG00000090554
CD8 T cells	CDKN2AIP	218929_at	55602	ENSG00000168564
CD8 T cells	TSC22D3	207001_x_at	1831	ENSG00000157514
CD8 T cells	TBCC	202495_at	6903	ENSG00000124659
CD8 T cells	RBM3	208319_s_at	5935	ENSG00000102317
CD8 T cells	ABT1	218405_at	29777	ENSG00000146109

CD8 T cells	C19orf6	212574_x_at	91304	ENSG00000182087
CD8 T cells	CAMLG	203538_at	819	ENSG00000164615
CD8 T cells	PPP1R2	202165_at	5504	ENSG00000184203
CD8 T cells	AES	217729_s_at	166	ENSG00000104964
CD8 T cells	KLF9	203543_s_at	687	ENSG00000119138
CD8 T cells	PRF1	214617_at	5551	ENSG00000180644
Tgd	TRD@	217143_s_at	6964	NaN
Tgd	TARP	211144_x_at	445347	ENSG00000211689
Tgd	C1orf61	205103_at	10485	ENSG00000125462
Tgd	TRGV9	209813_x_at	6983	NaN
Tgd	CD160	207840_at	11126	ENSG00000117281
Tgd	TARP	216920_s_at	445347	ENSG00000211689
Tgd	FEZ1	203562_at	9638	ENSG00000149557
Cytotoxic cells	KLRD1	210606_x_at	3824	ENSG00000134539
Cytotoxic cells	KLRF1	220646_s_at	51348	ENSG00000150045
Cytotoxic cells	GNLY	37145_at	10578	ENSG00000115523
Cytotoxic cells	GNLY	205495_s_at	10578	ENSG00000115523
Cytotoxic cells	CTSW	214450_at	1521	ENSG00000172543
Cytotoxic cells	KLRB1	214470_at	3820	ENSG00000111796
Cytotoxic cells	KLRD1	207795_s_at	3824	ENSG00000134539
Cytotoxic cells	KLRK1	205821_at	22914	ENSG00000213809
Cytotoxic cells	NKG7	213915_at	4818	ENSG00000105374
Cytotoxic cells	GZMH	210321_at	2999	ENSG00000100450
Cytotoxic cells	KLRD1	207796_x_at	3824	ENSG00000134539
Cytotoxic cells	SIGIRR	218921_at	59307	ENSG00000185187
Cytotoxic cells	ZBTB16	205883_at	7704	ENSG00000109906
Cytotoxic cells	RUNX3	204198_s_at	864	ENSG00000020633
Cytotoxic cells	APOL3	221087_s_at	80833	ENSG00000128284
Cytotoxic cells	RORA	210426_x_at	6095	ENSG00000069667
Cytotoxic cells	APBA2	209870_s_at	321	ENSG00000034053
Cytotoxic cells	APBA2	209870_s_at	321	ENSG00000276495
Cytotoxic cells	SIGIRR	52940_at	59307	ENSG00000185187
Cytotoxic cells	WHDC1L1	213908_at	339005	NaN
Cytotoxic cells	DUSP2	204794_at	1844	ENSG00000158050
Cytotoxic cells	GZMA	205488_at	3001	ENSG00000145649
NK cells	LOC643313	211050_x_at	643313	NaN
NK cells	GAGE2	207739_s_at	2574	ENSG00000236362
NK cells	ZNF747	206180_x_at	65988	ENSG00000169955
NK cells	XCL1	206366_x_at	6375	ENSG00000143184
NK cells	XCL2	214567_s_at	6846	ENSG00000143185
NK cells	AF107846	217058_at	2778	ENSG00000087460
NK cells	SLC30A5	220181_x_at	64924	ENSG00000145740
NK cells	NM_014114	220691_at	259230	ENSG00000198964
NK cells	MCM3AP	215582_x_at	8888	ENSG00000160294
NK cells	TBXA2R	207555_s_at	6915	ENSG00000006638
NK cells	CDC5L	209057_x_at	988	ENSG00000096401
NK cells	LOC730096	215182_x_at	730096	NaN
NK cells	FUT5	211225_at	2527	ENSG00000130383
NK cells	FGF18	206986_at	8817	ENSG00000156427
NK cells	MRC2	209280_at	9902	ENSG00000011028
NK cells	RP5-886K2.1	208014_x_at	27308	NaN
NK cells	SPN	216981_x_at	6693	ENSG00000197471
NK cells	PSMD4	210459_at	5710	ENSG00000159352
NK cells	PRX	220024_s_at	57716	ENSG00000105227

NK cells	FZR1	209415_at	51343	ENSG00000105325
NK cells	ZNF205	206416_at	7755	ENSG00000122386
NK cells	AL080130	212972_x_at	323	ENSG00000163697
NK cells	ZNF528	215019_x_at	84436	ENSG00000167555
NK cells	MAPRE3	203842_s_at	22924	ENSG00000084764
NK cells	BCL2	207004_at	596	ENSG00000171791
NK cells	NM_017616	221068_at	25959	ENSG00000197256
NK cells	ARL6IP2	217580_x_at	64225	ENSG00000119787
NK cells	SPN	206057_x_at	6693	ENSG00000197471
NK cells	FZR1	211865_s_at	51343	ENSG00000105325
NK cells	PDLIM4	214174_s_at	8572	ENSG00000131435
NK cells	NM_014274	206827_s_at	55503	ENSG00000165125
NK cells	NM_014274	206827_s_at	55503	ENSG00000276971
NK cells	LDB3	216888_at	11155	ENSG00000122367
NK cells	ADARB1	209979_at	104	ENSG00000197381
NK cells	SMEK1	215607_x_at	55671	ENSG00000100796
NK cells	TCTN2	206438_x_at	79867	ENSG00000168778
NK cells	TINAGL1	219058_x_at	64129	ENSG00000142910
NK cells	IGFBP5	203426_s_at	3488	ENSG00000115461
NK cells	ALDH1B1	209646_x_at	219	ENSG00000137124
NK cells	NCR1	217095_x_at	9437	ENSG00000278362
NK cells	NCR1	217095_x_at	9437	ENSG00000275156
NK cells	NCR1	217095_x_at	9437	ENSG00000277442
NK cells	NCR1	217095_x_at	9437	ENSG00000276450
NK cells	NCR1	217095_x_at	9437	ENSG00000278025
NK cells	NCR1	217095_x_at	9437	ENSG00000189430
NK cells	NCR1	217095_x_at	9437	ENSG00000275521
NK cells	NCR1	217095_x_at	9437	ENSG00000277334
NK cells	NCR1	217095_x_at	9437	ENSG00000275637
NK cells	NCR1	217095_x_at	9437	ENSG00000273535
NK cells	NCR1	217095_x_at	9437	ENSG00000277824
NK cells	NCR1	217095_x_at	9437	ENSG00000275822
NK cells	NCR1	217095_x_at	9437	ENSG00000274053
NK cells	NCR1	217095_x_at	9437	ENSG00000273506
NK cells	NCR1	217095_x_at	9437	ENSG00000277629
NK cells	NCR1	217095_x_at	9437	ENSG00000273916
NK cells	NCR1	217095_x_at	9437	ENSG00000284113
NK cells	NCR1	217095_x_at	9437	ENSG00000284208
NK cells	NCR1	217088_s_at	9437	ENSG00000278362
NK cells	NCR1	217088_s_at	9437	ENSG00000275156
NK cells	NCR1	217088_s_at	9437	ENSG00000277442
NK cells	NCR1	217088_s_at	9437	ENSG00000276450
NK cells	NCR1	217088_s_at	9437	ENSG00000278025
NK cells	NCR1	217088_s_at	9437	ENSG00000189430
NK cells	NCR1	217088_s_at	9437	ENSG00000275521
NK cells	NCR1	217088_s_at	9437	ENSG00000277334
NK cells	NCR1	217088_s_at	9437	ENSG00000275637
NK cells	NCR1	217088_s_at	9437	ENSG00000273535
NK cells	NCR1	217088_s_at	9437	ENSG00000277824
NK cells	NCR1	217088_s_at	9437	ENSG00000275822
NK cells	NCR1	217088_s_at	9437	ENSG00000274053
NK cells	NCR1	217088_s_at	9437	ENSG00000273506
NK cells	NCR1	217088_s_at	9437	ENSG00000277629
NK cells	NCR1	217088_s_at	9437	ENSG00000273916

NK cells	NCR1	217088_s_at	9437	ENSG00000284113
NK cells	NCR1	217088_s_at	9437	ENSG00000284208
NK cells	NCR1	207860_at	9437	ENSG00000278362
NK cells	NCR1	207860_at	9437	ENSG00000275156
NK cells	NCR1	207860_at	9437	ENSG00000277442
NK cells	NCR1	207860_at	9437	ENSG00000276450
NK cells	NCR1	207860_at	9437	ENSG00000278025
NK cells	NCR1	207860_at	9437	ENSG00000189430
NK cells	NCR1	207860_at	9437	ENSG00000275521
NK cells	NCR1	207860_at	9437	ENSG00000277334
NK cells	NCR1	207860_at	9437	ENSG00000275637
NK cells	NCR1	207860_at	9437	ENSG00000273535
NK cells	NCR1	207860_at	9437	ENSG00000277824
NK cells	NCR1	207860_at	9437	ENSG00000275822
NK cells	NCR1	207860_at	9437	ENSG00000274053
NK cells	NCR1	207860_at	9437	ENSG00000273506
NK cells	NCR1	207860_at	9437	ENSG00000277629
NK cells	NCR1	207860_at	9437	ENSG00000273916
NK cells	NCR1	207860_at	9437	ENSG00000284113
NK cells	NCR1	207860_at	9437	ENSG00000284208
NK CD56dim cells	KIR3DL2	207314_x_at	3812	ENSG00000278656
NK CD56dim cells	KIR3DL2	207314_x_at	3812	ENSG00000278361
NK CD56dim cells	KIR3DL2	207314_x_at	3812	ENSG00000277982
NK CD56dim cells	KIR3DL2	207314_x_at	3812	ENSG00000275626
NK CD56dim cells	KIR3DL2	207314_x_at	3812	ENSG00000276357
NK CD56dim cells	KIR3DL2	207314_x_at	3812	ENSG00000275511
NK CD56dim cells	KIR3DL2	207314_x_at	3812	ENSG00000273735
NK CD56dim cells	KIR3DL2	207314_x_at	3812	ENSG00000275262
NK CD56dim cells	KIR3DL2	207314_x_at	3812	ENSG00000278442
NK CD56dim cells	KIR3DL2	207314_x_at	3812	ENSG00000278707
NK CD56dim cells	KIR3DL2	207314_x_at	3812	ENSG00000278758
NK CD56dim cells	KIR3DL2	207314_x_at	3812	ENSG00000277181
NK CD56dim cells	KIR3DL2	207314_x_at	3812	ENSG00000275838
NK CD56dim cells	KIR3DL2	207314_x_at	3812	ENSG00000276739
NK CD56dim cells	KIR3DL2	207314_x_at	3812	ENSG00000277709
NK CD56dim cells	KIR3DL2	207314_x_at	3812	ENSG00000273911
NK CD56dim cells	KIR3DL2	207314_x_at	3812	ENSG00000276004
NK CD56dim cells	KIR3DL2	207314_x_at	3812	ENSG00000278403
NK CD56dim cells	KIR3DL2	207314_x_at	3812	ENSG00000275083
NK CD56dim cells	KIR3DL2	207314_x_at	3812	ENSG00000278809
NK CD56dim cells	KIR3DL2	207314_x_at	3812	ENSG00000278726
NK CD56dim cells	KIR3DL2	207314_x_at	3812	ENSG00000275629
NK CD56dim cells	KIR3DL2	207314_x_at	3812	ENSG00000276882
NK CD56dim cells	KIR3DL2	207314_x_at	3812	ENSG00000240403
NK CD56dim cells	KIR3DL2	207314_x_at	3812	ENSG00000278710
NK CD56dim cells	KIR3DL2	207314_x_at	3812	ENSG00000274722
NK CD56dim cells	KIR3DL2	207314_x_at	3812	ENSG00000275416
NK CD56dim cells	KIR3DL2	207314_x_at	3812	ENSG00000278474
NK CD56dim cells	KIR3DL2	207314_x_at	3812	ENSG00000275566
NK CD56dim cells	KIR3DL2	207314_x_at	3812	ENSG00000278850
NK CD56dim cells	KIR3DL2	207314_x_at	3812	ENSG00000276424
NK CD56dim cells	KIR3DL2	207314_x_at	3812	ENSG00000284295
NK CD56dim cells	KIR3DL2	207314_x_at	3812	ENSG00000284384
NK CD56dim cells	KIR3DL2	207314_x_at	3812	ENSG00000284466

NK CD56dim cells	KIR3DL2	207314_x_at	3812	ENSG00000284101
NK CD56dim cells	KIR3DL2	207314_x_at	3812	ENSG00000284213
NK CD56dim cells	KIR3DL2	207314_x_at	3812	ENSG00000284046
NK CD56dim cells	KIR3DL2	207314_x_at	3812	ENSG00000284063
NK CD56dim cells	KIR3DL2	207314_x_at	3812	ENSG00000283975
NK CD56dim cells	KIR3DL2	207314_x_at	3812	ENSG00000284528
NK CD56dim cells	KIR3DL2	207314_x_at	3812	ENSG00000284053
NK CD56dim cells	KIR3DL2	207314_x_at	3812	ENSG00000284192
NK CD56dim cells	KIR3DL2	207314_x_at	3812	ENSG00000283951
NK CD56dim cells	KIR3DL2	216907_x_at	3812	ENSG00000278656
NK CD56dim cells	KIR3DL2	216907_x_at	3812	ENSG00000278361
NK CD56dim cells	KIR3DL2	216907_x_at	3812	ENSG00000277982
NK CD56dim cells	KIR3DL2	216907_x_at	3812	ENSG00000275626
NK CD56dim cells	KIR3DL2	216907_x_at	3812	ENSG00000276357
NK CD56dim cells	KIR3DL2	216907_x_at	3812	ENSG00000275511
NK CD56dim cells	KIR3DL2	216907_x_at	3812	ENSG00000273735
NK CD56dim cells	KIR3DL2	216907_x_at	3812	ENSG00000275262
NK CD56dim cells	KIR3DL2	216907_x_at	3812	ENSG00000278442
NK CD56dim cells	KIR3DL2	216907_x_at	3812	ENSG00000278707
NK CD56dim cells	KIR3DL2	216907_x_at	3812	ENSG00000278758
NK CD56dim cells	KIR3DL2	216907_x_at	3812	ENSG00000277181
NK CD56dim cells	KIR3DL2	216907_x_at	3812	ENSG00000275838
NK CD56dim cells	KIR3DL2	216907_x_at	3812	ENSG00000276739
NK CD56dim cells	KIR3DL2	216907_x_at	3812	ENSG00000277709
NK CD56dim cells	KIR3DL2	216907_x_at	3812	ENSG00000273911
NK CD56dim cells	KIR3DL2	216907_x_at	3812	ENSG00000276004
NK CD56dim cells	KIR3DL2	216907_x_at	3812	ENSG00000278403
NK CD56dim cells	KIR3DL2	216907_x_at	3812	ENSG00000275083
NK CD56dim cells	KIR3DL2	216907_x_at	3812	ENSG00000278809
NK CD56dim cells	KIR3DL2	216907_x_at	3812	ENSG00000278726
NK CD56dim cells	KIR3DL2	216907_x_at	3812	ENSG00000275629
NK CD56dim cells	KIR3DL2	216907_x_at	3812	ENSG00000276882
NK CD56dim cells	KIR3DL2	216907_x_at	3812	ENSG00000240403
NK CD56dim cells	KIR3DL2	216907_x_at	3812	ENSG00000278710
NK CD56dim cells	KIR3DL2	216907_x_at	3812	ENSG00000274722
NK CD56dim cells	KIR3DL2	216907_x_at	3812	ENSG00000275416
NK CD56dim cells	KIR3DL2	216907_x_at	3812	ENSG00000278474
NK CD56dim cells	KIR3DL2	216907_x_at	3812	ENSG00000275566
NK CD56dim cells	KIR3DL2	216907_x_at	3812	ENSG00000278850
NK CD56dim cells	KIR3DL2	216907_x_at	3812	ENSG00000276424
NK CD56dim cells	KIR3DL2	216907_x_at	3812	ENSG00000284295
NK CD56dim cells	KIR3DL2	216907_x_at	3812	ENSG00000284384
NK CD56dim cells	KIR3DL2	216907_x_at	3812	ENSG00000284466
NK CD56dim cells	KIR3DL2	216907_x_at	3812	ENSG00000284101
NK CD56dim cells	KIR3DL2	216907_x_at	3812	ENSG00000284213
NK CD56dim cells	KIR3DL2	216907_x_at	3812	ENSG00000284046
NK CD56dim cells	KIR3DL2	216907_x_at	3812	ENSG00000284063
NK CD56dim cells	KIR3DL2	216907_x_at	3812	ENSG00000283975
NK CD56dim cells	KIR3DL2	216907_x_at	3812	ENSG00000284528
NK CD56dim cells	KIR3DL2	216907_x_at	3812	ENSG00000284053
NK CD56dim cells	KIR3DL2	216907_x_at	3812	ENSG00000284192
NK CD56dim cells	KIR3DL2	216907_x_at	3812	ENSG00000283951
NK CD56dim cells	SPON2	218638_s_at	10417	ENSG00000159674
NK CD56dim cells	KIR2DL3	208179_x_at	3804	ENSG00000275623

NK CD56dim cells	KIR2DL3	208179_x_at	3804	ENSG00000275008
NK CD56dim cells	KIR2DL3	208179_x_at	3804	ENSG00000274952
NK CD56dim cells	KIR2DL3	208179_x_at	3804	ENSG00000276459
NK CD56dim cells	KIR2DL3	208179_x_at	3804	ENSG00000277554
NK CD56dim cells	KIR2DL3	208179_x_at	3804	ENSG00000276590
NK CD56dim cells	KIR2DL3	208179_x_at	3804	ENSG00000274410
NK CD56dim cells	KIR2DL3	208179_x_at	3804	ENSG00000275658
NK CD56dim cells	KIR2DL3	208179_x_at	3804	ENSG00000274830
NK CD56dim cells	KIR2DL3	208179_x_at	3804	ENSG00000278327
NK CD56dim cells	KIR2DL3	208179_x_at	3804	ENSG00000274402
NK CD56dim cells	KIR2DL3	208179_x_at	3804	ENSG00000277484
NK CD56dim cells	KIR2DL3	208179_x_at	3804	ENSG00000277317
NK CD56dim cells	KIR2DL3	208179_x_at	3804	ENSG00000273887
NK CD56dim cells	KIR2DL3	208179_x_at	3804	ENSG00000243772
NK CD56dim cells	KIR2DL3	208179_x_at	3804	ENSG00000274108
NK CD56dim cells	KIR2DL3	208179_x_at	3804	ENSG00000273947
NK CD56dim cells	KIR2DL3	208179_x_at	3804	ENSG00000277924
NK CD56dim cells	KIR2DL3	208179_x_at	3804	ENSG00000278369
NK CD56dim cells	KIR2DL3	208179_x_at	3804	ENSG00000276218
NK CD56dim cells	KIR2DL3	208179_x_at	3804	ENSG00000284132
NK CD56dim cells	KIR2DL3	208179_x_at	3804	ENSG00000284504
NK CD56dim cells	KIR2DL3	208179_x_at	3804	ENSG00000284510
NK CD56dim cells	KIR2DL3	208179_x_at	3804	ENSG00000283996
NK CD56dim cells	KIR2DL3	208179_x_at	3804	ENSG00000283708
NK CD56dim cells	KIR2DL3	208179_x_at	3804	ENSG00000283702
NK CD56dim cells	KIR2DL3	208179_x_at	3804	ENSG00000283790
NK CD56dim cells	KIR2DL3	208179_x_at	3804	ENSG00000284236
NK CD56dim cells	KIR2DL3	208179_x_at	3804	ENSG00000284044
NK CD56dim cells	KIR2DL3	208179_x_at	3804	ENSG00000284333
NK CD56dim cells	KIR2DL3	208179_x_at	3804	ENSG00000284241
NK CD56dim cells	GZMB	210164_at	3002	ENSG00000100453
NK CD56dim cells	KIR3DS1	211389_x_at	3813	ENSG00000274283
NK CD56dim cells	KIR3DS1	211389_x_at	3813	ENSG00000275608
NK CD56dim cells	KIR3DS1	211389_x_at	3813	ENSG00000276534
NK CD56dim cells	KIR3DS1	211389_x_at	3813	ENSG00000275434
NK CD56dim cells	KIR3DS1	211389_x_at	3813	ENSG00000276498
NK CD56dim cells	KIR3DS1	211389_x_at	3813	ENSG00000275037
NK CD56dim cells	KIR3DS1	211389_x_at	3813	ENSG00000274465
NK CD56dim cells	KIR3DL1	211687_x_at	3811	ENSG00000274146
NK CD56dim cells	KIR3DL1	211687_x_at	3811	ENSG00000277175
NK CD56dim cells	KIR3DL1	211687_x_at	3811	ENSG00000274920
NK CD56dim cells	KIR3DL1	211687_x_at	3811	ENSG00000278427
NK CD56dim cells	KIR3DL1	211687_x_at	3811	ENSG00000278856
NK CD56dim cells	KIR3DL1	211687_x_at	3811	ENSG00000273518
NK CD56dim cells	KIR3DL1	211687_x_at	3811	ENSG00000276501
NK CD56dim cells	KIR3DL1	211687_x_at	3811	ENSG00000277272
NK CD56dim cells	KIR3DL1	211687_x_at	3811	ENSG00000274036
NK CD56dim cells	KIR3DL1	211687_x_at	3811	ENSG00000274948
NK CD56dim cells	KIR3DL1	211687_x_at	3811	ENSG00000275717
NK CD56dim cells	KIR3DL1	211687_x_at	3811	ENSG00000275486
NK CD56dim cells	KIR3DL1	211687_x_at	3811	ENSG00000276379
NK CD56dim cells	KIR3DL1	211687_x_at	3811	ENSG00000275288
NK CD56dim cells	KIR3DL1	211687_x_at	3811	ENSG00000275786
NK CD56dim cells	KIR3DL1	211687_x_at	3811	ENSG00000167633

NK CD56dim cells	KIR3DL1	211687_x_at	3811	ENSG00000278368
NK CD56dim cells	KIR3DL1	211687_x_at	3811	ENSG00000276423
NK CD56dim cells	KIR3DL1	211687_x_at	3811	ENSG00000278079
NK CD56dim cells	KIR3DL1	211687_x_at	3811	ENSG00000275545
NK CD56dim cells	KIR3DL1	211687_x_at	3811	ENSG00000275659
NK CD56dim cells	KIR3DL1	211687_x_at	3811	ENSG00000273775
NK CD56dim cells	KIR3DL1	211687_x_at	3811	ENSG00000276329
NK CD56dim cells	KIR3DL1	211687_x_at	3811	ENSG00000284426
NK CD56dim cells	KIR3DL1	211687_x_at	3811	ENSG00000283954
NK CD56dim cells	KIR3DL1	211687_x_at	3811	ENSG00000283729
NK CD56dim cells	KIR3DL1	211687_x_at	3811	ENSG00000284589
NK CD56dim cells	KIR3DL1	211687_x_at	3811	ENSG00000284177
NK CD56dim cells	KIR3DL1	211687_x_at	3811	ENSG00000283731
NK CD56dim cells	KIR3DL1	211687_x_at	3811	ENSG00000284093
NK CD56dim cells	KIR3DL1	211687_x_at	3811	ENSG00000283827
NK CD56dim cells	FLJ20699	216434_at	55020	ENSG00000075234
NK CD56dim cells	TMEPAI	217875_s_at	56937	ENSG00000124225
NK CD56dim cells	KIR3DL2	211688_x_at	3812	ENSG00000278656
NK CD56dim cells	KIR3DL2	211688_x_at	3812	ENSG00000278361
NK CD56dim cells	KIR3DL2	211688_x_at	3812	ENSG00000277982
NK CD56dim cells	KIR3DL2	211688_x_at	3812	ENSG00000275626
NK CD56dim cells	KIR3DL2	211688_x_at	3812	ENSG00000276357
NK CD56dim cells	KIR3DL2	211688_x_at	3812	ENSG00000275511
NK CD56dim cells	KIR3DL2	211688_x_at	3812	ENSG00000273735
NK CD56dim cells	KIR3DL2	211688_x_at	3812	ENSG00000275262
NK CD56dim cells	KIR3DL2	211688_x_at	3812	ENSG00000278442
NK CD56dim cells	KIR3DL2	211688_x_at	3812	ENSG00000278707
NK CD56dim cells	KIR3DL2	211688_x_at	3812	ENSG00000278758
NK CD56dim cells	KIR3DL2	211688_x_at	3812	ENSG00000277181
NK CD56dim cells	KIR3DL2	211688_x_at	3812	ENSG00000275838
NK CD56dim cells	KIR3DL2	211688_x_at	3812	ENSG00000276739
NK CD56dim cells	KIR3DL2	211688_x_at	3812	ENSG00000277709
NK CD56dim cells	KIR3DL2	211688_x_at	3812	ENSG00000273911
NK CD56dim cells	KIR3DL2	211688_x_at	3812	ENSG00000276004
NK CD56dim cells	KIR3DL2	211688_x_at	3812	ENSG00000278403
NK CD56dim cells	KIR3DL2	211688_x_at	3812	ENSG00000275083
NK CD56dim cells	KIR3DL2	211688_x_at	3812	ENSG00000278809
NK CD56dim cells	KIR3DL2	211688_x_at	3812	ENSG00000278726
NK CD56dim cells	KIR3DL2	211688_x_at	3812	ENSG00000275629
NK CD56dim cells	KIR3DL2	211688_x_at	3812	ENSG00000276882
NK CD56dim cells	KIR3DL2	211688_x_at	3812	ENSG00000240403
NK CD56dim cells	KIR3DL2	211688_x_at	3812	ENSG00000278710
NK CD56dim cells	KIR3DL2	211688_x_at	3812	ENSG00000274722
NK CD56dim cells	KIR3DL2	211688_x_at	3812	ENSG00000275416
NK CD56dim cells	KIR3DL2	211688_x_at	3812	ENSG00000278474
NK CD56dim cells	KIR3DL2	211688_x_at	3812	ENSG00000275566
NK CD56dim cells	KIR3DL2	211688_x_at	3812	ENSG00000278850
NK CD56dim cells	KIR3DL2	211688_x_at	3812	ENSG00000276424
NK CD56dim cells	KIR3DL2	211688_x_at	3812	ENSG00000284295
NK CD56dim cells	KIR3DL2	211688_x_at	3812	ENSG00000284384
NK CD56dim cells	KIR3DL2	211688_x_at	3812	ENSG00000284466
NK CD56dim cells	KIR3DL2	211688_x_at	3812	ENSG00000284101
NK CD56dim cells	KIR3DL2	211688_x_at	3812	ENSG00000284213
NK CD56dim cells	KIR3DL2	211688_x_at	3812	ENSG00000284046

NK CD56dim cells	KIR3DL2	211688_x_at	3812	ENSG00000284063
NK CD56dim cells	KIR3DL2	211688_x_at	3812	ENSG00000283975
NK CD56dim cells	KIR3DL2	211688_x_at	3812	ENSG00000284528
NK CD56dim cells	KIR3DL2	211688_x_at	3812	ENSG00000284053
NK CD56dim cells	KIR3DL2	211688_x_at	3812	ENSG00000284192
NK CD56dim cells	KIR3DL2	211688_x_at	3812	ENSG00000283951
NK CD56dim cells	IL21R	221658_s_at	50615	ENSG00000103522
NK CD56dim cells	KIR3DL2	207313_x_at	3812	ENSG00000278656
NK CD56dim cells	KIR3DL2	207313_x_at	3812	ENSG00000278361
NK CD56dim cells	KIR3DL2	207313_x_at	3812	ENSG00000277982
NK CD56dim cells	KIR3DL2	207313_x_at	3812	ENSG00000275626
NK CD56dim cells	KIR3DL2	207313_x_at	3812	ENSG00000276357
NK CD56dim cells	KIR3DL2	207313_x_at	3812	ENSG00000275511
NK CD56dim cells	KIR3DL2	207313_x_at	3812	ENSG00000273735
NK CD56dim cells	KIR3DL2	207313_x_at	3812	ENSG00000275262
NK CD56dim cells	KIR3DL2	207313_x_at	3812	ENSG00000278442
NK CD56dim cells	KIR3DL2	207313_x_at	3812	ENSG00000278707
NK CD56dim cells	KIR3DL2	207313_x_at	3812	ENSG00000278758
NK CD56dim cells	KIR3DL2	207313_x_at	3812	ENSG00000277181
NK CD56dim cells	KIR3DL2	207313_x_at	3812	ENSG00000275838
NK CD56dim cells	KIR3DL2	207313_x_at	3812	ENSG00000276739
NK CD56dim cells	KIR3DL2	207313_x_at	3812	ENSG00000277709
NK CD56dim cells	KIR3DL2	207313_x_at	3812	ENSG00000273911
NK CD56dim cells	KIR3DL2	207313_x_at	3812	ENSG00000276004
NK CD56dim cells	KIR3DL2	207313_x_at	3812	ENSG00000278403
NK CD56dim cells	KIR3DL2	207313_x_at	3812	ENSG00000275083
NK CD56dim cells	KIR3DL2	207313_x_at	3812	ENSG00000278809
NK CD56dim cells	KIR3DL2	207313_x_at	3812	ENSG00000278726
NK CD56dim cells	KIR3DL2	207313_x_at	3812	ENSG00000275629
NK CD56dim cells	KIR3DL2	207313_x_at	3812	ENSG00000276882
NK CD56dim cells	KIR3DL2	207313_x_at	3812	ENSG00000240403
NK CD56dim cells	KIR3DL2	207313_x_at	3812	ENSG00000278710
NK CD56dim cells	KIR3DL2	207313_x_at	3812	ENSG00000274722
NK CD56dim cells	KIR3DL2	207313_x_at	3812	ENSG00000275416
NK CD56dim cells	KIR3DL2	207313_x_at	3812	ENSG00000278474
NK CD56dim cells	KIR3DL2	207313_x_at	3812	ENSG00000275566
NK CD56dim cells	KIR3DL2	207313_x_at	3812	ENSG00000278850
NK CD56dim cells	KIR3DL2	207313_x_at	3812	ENSG00000276424
NK CD56dim cells	KIR3DL2	207313_x_at	3812	ENSG00000284295
NK CD56dim cells	KIR3DL2	207313_x_at	3812	ENSG00000284384
NK CD56dim cells	KIR3DL2	207313_x_at	3812	ENSG00000284466
NK CD56dim cells	KIR3DL2	207313_x_at	3812	ENSG00000284101
NK CD56dim cells	KIR3DL2	207313_x_at	3812	ENSG00000284213
NK CD56dim cells	KIR3DL2	207313_x_at	3812	ENSG00000284046
NK CD56dim cells	KIR3DL2	207313_x_at	3812	ENSG00000284063
NK CD56dim cells	KIR3DL2	207313_x_at	3812	ENSG00000283975
NK CD56dim cells	KIR3DL2	207313_x_at	3812	ENSG00000284528
NK CD56dim cells	KIR3DL2	207313_x_at	3812	ENSG00000284053
NK CD56dim cells	KIR3DL2	207313_x_at	3812	ENSG00000284192
NK CD56dim cells	KIR3DL2	207313_x_at	3812	ENSG00000283951
NK CD56dim cells	KIR3DL3	216676_x_at	115653	ENSG00000278490
NK CD56dim cells	KIR3DL3	216676_x_at	115653	ENSG00000274763
NK CD56dim cells	KIR3DL3	216676_x_at	115653	ENSG00000275513
NK CD56dim cells	KIR3DL3	216676_x_at	115653	ENSG00000275062

NK CD56dim cells	KIR3DL3	216676_x_at	115653	ENSG00000278723
NK CD56dim cells	KIR3DL3	216676_x_at	115653	ENSG00000274254
NK CD56dim cells	KIR3DL3	216676_x_at	115653	ENSG00000274639
NK CD56dim cells	KIR3DL3	216676_x_at	115653	ENSG00000276086
NK CD56dim cells	KIR3DL3	216676_x_at	115653	ENSG00000274511
NK CD56dim cells	KIR3DL3	216676_x_at	115653	ENSG00000276433
NK CD56dim cells	KIR3DL3	216676_x_at	115653	ENSG00000276328
NK CD56dim cells	KIR3DL3	216676_x_at	115653	ENSG00000277620
NK CD56dim cells	KIR3DL3	216676_x_at	115653	ENSG00000274786
NK CD56dim cells	KIR3DL3	216676_x_at	115653	ENSG00000276196
NK CD56dim cells	KIR3DL3	216676_x_at	115653	ENSG00000275172
NK CD56dim cells	KIR3DL3	216676_x_at	115653	ENSG00000276572
NK CD56dim cells	KIR3DL3	216676_x_at	115653	ENSG00000276875
NK CD56dim cells	KIR3DL3	216676_x_at	115653	ENSG00000274556
NK CD56dim cells	KIR3DL3	216676_x_at	115653	ENSG00000276806
NK CD56dim cells	KIR3DL3	216676_x_at	115653	ENSG00000276084
NK CD56dim cells	KIR3DL3	216676_x_at	115653	ENSG00000274394
NK CD56dim cells	KIR3DL3	216676_x_at	115653	ENSG00000274696
NK CD56dim cells	KIR3DL3	216676_x_at	115653	ENSG00000276930
NK CD56dim cells	KIR3DL3	216676_x_at	115653	ENSG00000277028
NK CD56dim cells	KIR3DL3	216676_x_at	115653	ENSG00000277552
NK CD56dim cells	KIR3DL3	216676_x_at	115653	ENSG00000242019
NK CD56dim cells	KIR3DL3	216676_x_at	115653	ENSG00000277392
NK CD56dim cells	KIR3DL3	216676_x_at	115653	ENSG00000274480
NK CD56dim cells	KIR3DL3	216676_x_at	115653	ENSG00000275433
NK CD56dim cells	KIR3DL3	216676_x_at	115653	ENSG00000278729
NK CD56dim cells	KIR3DL3	216676_x_at	115653	ENSG00000277596
NK CD56dim cells	KIR3DL3	216676_x_at	115653	ENSG00000273502
NK CD56dim cells	KIR3DL3	216676_x_at	115653	ENSG00000274724
NK CD56dim cells	KIR3DL3	216676_x_at	115653	ENSG00000283875
NK CD56dim cells	KIR3DL3	216676_x_at	115653	ENSG00000284104
NK CD56dim cells	KIR3DL3	216676_x_at	115653	ENSG00000284371
NK CD56dim cells	KIR3DL3	216676_x_at	115653	ENSG00000283823
NK CD56dim cells	KIR3DL3	216676_x_at	115653	ENSG00000283915
NK CD56dim cells	KIR3DL3	216676_x_at	115653	ENSG00000284086
NK CD56dim cells	KIR3DL3	216676_x_at	115653	ENSG00000283966
NK CD56dim cells	KIR3DL3	216676_x_at	115653	ENSG00000284480
NK CD56dim cells	KIR3DL3	216676_x_at	115653	ENSG00000284127
NK CD56dim cells	KIR2DS5	208203_x_at	3810	ENSG00000274739
NK CD56dim cells	KIR2DS5	208203_x_at	3810	ENSG00000276676
NK CD56dim cells	KIR2DS2	211532_x_at	3807	NaN
NK CD56dim cells	GTF3C1	202320_at	2975	ENSG00000077235
NK CD56dim cells	KIR2DS1	216552_x_at	3806	ENSG00000278304
NK CD56dim cells	KIR2DS1	216552_x_at	3806	ENSG00000273603
NK CD56dim cells	KIR2DS1	216552_x_at	3806	ENSG00000273517
NK CD56dim cells	KIR2DS1	216552_x_at	3806	ENSG00000278120
NK CD56dim cells	KIR2DS1	216552_x_at	3806	ENSG00000275421
NK CD56dim cells	KIR2DS1	216552_x_at	3806	ENSG00000275306
NK CD56dim cells	KIR2DS1	216552_x_at	3806	ENSG00000276327
NK CD56dim cells	EDG8	221417_x_at	53637	ENSG00000180739
NK CD56bright cells	DUSP4	204014_at	1846	ENSG00000120875
NK CD56bright cells	RRAD	204803_s_at	6236	ENSG00000166592
NK CD56bright cells	XCL1	206365_at	6375	ENSG00000143184
NK CD56bright cells	PLA2G6	215938_s_at	8398	ENSG00000184381

NK CD56bright cells	PLA2G6	204691_x_at	8398	ENSG00000184381
NK CD56bright cells	NIBP	221672_s_at	83696	ENSG00000167632
NK CD56bright cells	FOXJ1	205906_at	2302	ENSG00000129654
NK CD56bright cells	03/06/09	215908_at	10299	ENSG00000145495
NK CD56bright cells	DUSP4	204015_s_at	1846	ENSG00000120875
NK CD56bright cells	PLA2G6	210647_x_at	8398	ENSG00000184381
NK CD56bright cells	MADD	38398_at	8567	ENSG00000110514
NK CD56bright cells	BG255923	215409_at	254531	ENSG00000176454
NK CD56bright cells	MPPED1	206436_at	758	ENSG00000186732
NK CD56bright cells	MUC3B	214676_x_at	57876	NaN
DC	CD209	207277_at	30835	ENSG00000090659
DC	CCL17	207900_at	6361	ENSG00000102970
DC	HSD11B1	205404_at	3290	ENSG00000117594
DC	CCL13	206407_s_at	6357	ENSG00000181374
DC	CCL22	207861_at	6367	ENSG00000102962
DC	PPFIBP2	212841_s_at	8495	ENSG00000166387
DC	NPR1	32625_at	4881	ENSG00000169418
iDC	CD1B	206749_at	910	ENSG00000158485
iDC	VASH1	203940_s_at	22846	ENSG00000071246
iDC	F13A1	203305_at	2162	ENSG00000124491
iDC	CD1E	215784_at	913	ENSG00000158488
iDC	MMP12	204580_at	4321	ENSG00000262406
iDC	FABP4	203980_at	2167	ENSG00000170323
iDC	CLEC10A	206682_at	10462	ENSG00000132514
iDC	SYT17	205613_at	51760	ENSG00000103528
iDC	MS4A6A	219666_at	64231	ENSG00000110077
iDC	CTNS	204925_at	1497	ENSG00000040531
iDC	GUCA1A	206062_at	2978	ENSG00000048545
iDC	CARD9	220162_s_at	64170	ENSG00000187796
iDC	CD1E	208592_s_at	913	ENSG00000158488
iDC	ABCG2	209735_at	9429	ENSG00000118777
iDC	CD1A	210325_at	909	ENSG00000158477
iDC	PPARG	208510_s_at	5468	ENSG00000132170
iDC	RAP1GAP	203911_at	5909	ENSG00000076864
iDC	SLC7A8	216604_s_at	23428	ENSG00000092068
iDC	GSTT1	203815_at	2952	ENSG00000277656
iDC	NM_021941	218019_s_at	8566	ENSG00000160209
iDC	FZD2	210220_at	2535	ENSG00000180340
iDC	CSF1R	203104_at	1436	ENSG00000182578
iDC	HS3ST2	219697_at	9956	ENSG00000122254
iDC	CH25H	206932_at	9023	ENSG00000138135
iDC	LMAN2L	221274_s_at	81562	ENSG00000114988
iDC	SLC26A6	221572_s_at	65010	ENSG00000225697
iDC	BLVRB	202201_at	645	ENSG00000090013
iDC	NUDT9	218375_at	53343	ENSG00000170502
iDC	PREP	204117_at	5550	ENSG00000085377
iDC	TM7SF4	221266_s_at	81501	ENSG00000164935
iDC	TACSTD2	202286_s_at	4070	ENSG00000184292
iDC	CD1C	205987_at	911	ENSG00000158481
aDC	CCL1	207533_at	6346	ENSG00000108702
aDC	EBI3	219424_at	10148	ENSG00000105246
aDC	INDO	210029_at	3620	ENSG00000131203
aDC	LAMP3	205569_at	27074	ENSG00000078081
aDC	OAS3	218400_at	4940	ENSG00000111331

pDC	IL3RA	206148_at	3563	ENSG00000185291
Eosinophils	IL5RA	211517_s_at	3568	ENSG00000091181
Eosinophils	KCNH2	205262_at	3757	ENSG00000055118
Eosinophils	TKTL1	216370_s_at	8277	ENSG00000007350
Eosinophils	IL5RA	210744_s_at	3568	ENSG00000091181
Eosinophils	EMR1	207111_at	2015	ENSG00000174837
Eosinophils	KCNH2	210036_s_at	3757	ENSG00000055118
Eosinophils	CCR3	208304_at	1232	ENSG00000183625
Eosinophils	ACACB	49452_at	32	ENSG00000076555
Eosinophils	THBS1	201108_s_at	7057	ENSG00000137801
Eosinophils	GALC	211810_s_at	2581	ENSG00000054983
Eosinophils	TKTL1	214183_s_at	8277	ENSG00000007350
Eosinophils	RNU2	210230_at	728965	NaN
Eosinophils	CLC	206207_at	1178	ENSG00000105205
Eosinophils	THBS1	201109_s_at	7057	ENSG00000137801
Eosinophils	HIST1H1C	209398_at	3006	ENSG00000187837
Eosinophils	CYSLTR2	220813_at	57105	ENSG00000152207
Eosinophils	HRH4	221170_at	59340	ENSG00000134489
Eosinophils	RNASE2	206111_at	6036	ENSG00000169385
Eosinophils	CAT	211922_s_at	847	ENSG00000121691
Eosinophils	LRP5L	214873_at	91355	ENSG00000100068
Eosinophils	SYNJ1	207594_s_at	8867	ENSG00000159082
Eosinophils	SYNJ1	212990_at	8867	ENSG00000159082
Eosinophils	THBS4	204776_at	7060	ENSG00000113296
Eosinophils	GPR44	206361_at	11251	ENSG00000183134
Eosinophils	KBTBD11	204301_at	9920	ENSG00000176595
Eosinophils	KBTBD11	204301_at	9920	ENSG00000273645
Eosinophils	HES1	203394_s_at	3280	ENSG00000114315
Eosinophils	ABHD2	205566_at	11057	ENSG00000140526
Eosinophils	TIPARP	212665_at	25976	ENSG00000163659
Eosinophils	SMPD3	219695_at	55512	ENSG00000103056
Eosinophils	MYO15B	59375_at	80022	NaN
Eosinophils	TGIF1	203313_s_at	7050	ENSG00000177426
Eosinophils	RRP12	216360_x_at	23223	ENSG00000052749
Eosinophils	ACACB	43427_at	32	ENSG00000076555
Eosinophils	IGSF2	207167_at	9398	ENSG00000134256
Eosinophils	HES1	203395_s_at	3280	ENSG00000114315
Eosinophils	RCOR3	218344_s_at	55758	ENSG00000117625
Eosinophils	EPN2	203463_s_at	22905	ENSG00000072134
Eosinophils	C9orf156	222195_s_at	51531	ENSG00000136932
Eosinophils	SIAH1	202981_x_at	6477	ENSG00000196470
Eosinophils	ACACB	221928_at	32	ENSG00000076555
Macrophages	MARCO	205819_at	8685	ENSG00000019169
Macrophages	CXCL5	214974_x_at	6374	ENSG00000163735
Macrophages	SCG5	203889_at	6447	ENSG00000166922
Macrophages	SCG5	203889_at	6447	ENSG00000277614
Macrophages	SCG5	203889_at	6447	ENSG00000281931
Macrophages	SULT1C2	205342_s_at	6819	ENSG00000198203
Macrophages	SULT1C2	211470_s_at	6819	ENSG00000198203
Macrophages	MSR1	214770_at	4481	ENSG00000038945
Macrophages	CTSK	202450_s_at	1513	ENSG00000143387
Macrophages	PTGDS	212187_x_at	5730	ENSG00000107317
Macrophages	COLEC12	221019_s_at	81035	ENSG00000158270
Macrophages	GPC4	204984_at	2239	ENSG00000076716

Macrophages	MSR1	208423_s_at	4481	ENSG00000038945
Macrophages	PCOLCE2	219295_s_at	26577	ENSG00000163710
Macrophages	CHIT1	208168_s_at	1118	ENSG00000133063
Macrophages	PTGDS	211748_x_at	5730	ENSG00000107317
Macrophages	KAL1	205206_at	3730	ENSG00000011201
Macrophages	CLEC5A	219890_at	23601	ENSG00000258227
Macrophages	GPC4	204983_s_at	2239	ENSG00000076716
Macrophages	ME1	204058_at	4199	ENSG00000065833
Macrophages	DNASE2B	220380_at	58511	ENSG00000137976
Macrophages	CCL7	208075_s_at	6354	ENSG00000108688
Macrophages	FN1	214701_s_at	2335	ENSG00000115414
Macrophages	CD163	203645_s_at	9332	ENSG00000177575
Macrophages	GM2A	215891_s_at	2760	ENSG00000196743
Macrophages	SCARB2	201647_s_at	950	ENSG00000138760
Macrophages	BCAT1	214452_at	586	ENSG00000060982
Macrophages	BCAT1	214390_s_at	586	ENSG00000060982
Macrophages	RAI14	202052_s_at	26064	ENSG00000039560
Macrophages	MSR1	211887_x_at	4481	ENSG00000038945
Macrophages	COL8A2	52651_at	1296	ENSG00000171812
Macrophages	CD163	215049_x_at	9332	ENSG00000177575
Macrophages	APOE	203381_s_at	348	ENSG00000130203
Macrophages	CHI3L1	209396_s_at	1116	ENSG00000133048
Macrophages	ATG7	218673_s_at	10533	ENSG00000197548
Macrophages	CD84	211190_x_at	8832	ENSG00000066294
Macrophages	FDX1	203646_at	2230	ENSG00000137714
Macrophages	MS4A4A	219607_s_at	51338	ENSG00000110079
Macrophages	SGMS1	212989_at	259230	ENSG00000198964
Macrophages	EMP1	201324_at	2012	ENSG00000134531
Macrophages	CYBB	203922_s_at	1536	ENSG00000165168
Macrophages	CD68	203507_at	968	ENSG00000129226
Mast cells	PRG2	211743_s_at	5553	ENSG00000186652
Mast cells	CTSG	205653_at	1511	ENSG00000100448
Mast cells	TPSAB1	215382_x_at	7177	ENSG00000172236
Mast cells	SLC18A2	205857_at	6571	ENSG00000165646
Mast cells	TPSAB1	205683_x_at	7177	ENSG00000172236
Mast cells	MS4A2	207497_s_at	2206	ENSG00000149534
Mast cells	CPA3	205624_at	1359	ENSG00000163751
Mast cells	TPSB2	207134_x_at	64499	ENSG00000197253
Mast cells	TPSAB1	216474_x_at	7177	ENSG00000172236
Mast cells	NM_003293	207741_x_at	64499	ENSG00000197253
Mast cells	TPSAB1	210084_x_at	7177	ENSG00000172236
Mast cells	MS4A2	207496_at	2206	ENSG00000149534
Mast cells	TPSAB1	217023_x_at	7177	ENSG00000172236
Mast cells	GATA2	209710_at	2624	ENSG00000179348
Mast cells	HDC	207067_s_at	3067	ENSG00000140287
Mast cells	LOH11CR2A	210102_at	4013	ENSG00000110002
Mast cells	SIGLEC6	210796_x_at	946	ENSG00000105492
Mast cells	ELA2	206871_at	1991	ENSG00000277571
Mast cells	ELA2	206871_at	1991	ENSG00000197561
Mast cells	LOH11CR2A	205011_at	4013	ENSG00000110002
Mast cells	CMA1	214533_at	1215	ENSG00000092009
Mast cells	SIGLEC6	206520_x_at	946	ENSG00000105492
Mast cells	PGDS	206726_at	27306	ENSG00000163106
Mast cells	MLPH	218211_s_at	79083	ENSG00000115648

Mast cells	ADCYAP1	206281_at	116	ENSG00000141433
Mast cells	SIGLEC6	206519_x_at	946	ENSG00000105492
Mast cells	SLC24A3	57588_at	57419	ENSG00000185052
Mast cells	CALB2	205428_s_at	794	ENSG00000172137
Mast cells	CALB2	205428_s_at	794	ENSG00000282830
Mast cells	SLC24A3	219090_at	57419	ENSG00000185052
Mast cells	KIT	205051_s_at	3815	ENSG00000157404
Mast cells	TAL1	206283_s_at	6886	ENSG00000162367
Mast cells	ABCC4	203196_at	10257	ENSG00000125257
Mast cells	PPM1H	212686_at	57460	ENSG00000111110
Mast cells	MAOB	204041_at	4129	ENSG00000069535
Mast cells	HPGD	211549_s_at	3248	ENSG00000164120
Mast cells	SCG2	204035_at	7857	ENSG00000171951
Mast cells	PTGS1	205127_at	5742	ENSG00000095303
Mast cells	CEACAM8	206676_at	1088	ENSG00000124469
Mast cells	MPO	203949_at	4353	ENSG00000005381
Mast cells	NR0B1	206645_s_at	190	ENSG00000169297
Mast cells	LOC339524	215039_at	339524	ENSG00000267272
Neutrophils	CSF3R	203591_s_at	1441	ENSG00000119535
Neutrophils	CYP4F3	206515_at	4051	ENSG00000186529
Neutrophils	VNN3	220528_at	55350	ENSG00000093134
Neutrophils	FPRL1	210773_s_at	2358	ENSG00000171049
Neutrophils	KCNJ15	216782_at	3772	ENSG00000157551
Neutrophils	MME	203434_s_at	4311	ENSG00000196549
Neutrophils	IL8RA	207094_at	3577	ENSG00000163464
Neutrophils	IL8RB	207008_at	3579	ENSG00000180871
Neutrophils	MME	203435_s_at	4311	ENSG00000196549
Neutrophils	FCGR3B	204007_at	2215	ENSG00000162747
Neutrophils	DYSF	218660_at	8291	ENSG00000135636
Neutrophils	KCNJ15	211806_s_at	3772	ENSG00000157551
Neutrophils	FCAR	211816_x_at	2204	ENSG00000278415
Neutrophils	FCAR	211816_x_at	2204	ENSG00000274580
Neutrophils	FCAR	211816_x_at	2204	ENSG00000275136
Neutrophils	FCAR	211816_x_at	2204	ENSG00000275970
Neutrophils	FCAR	211816_x_at	2204	ENSG00000276985
Neutrophils	FCAR	211816_x_at	2204	ENSG00000186431
Neutrophils	FCAR	211816_x_at	2204	ENSG00000276858
Neutrophils	FCAR	211816_x_at	2204	ENSG00000275269
Neutrophils	FCAR	211816_x_at	2204	ENSG00000273738
Neutrophils	FCAR	211816_x_at	2204	ENSG00000275564
Neutrophils	FCAR	211816_x_at	2204	ENSG00000283953
Neutrophils	FCAR	211816_x_at	2204	ENSG00000283750
Neutrophils	FCAR	211816_x_at	2204	ENSG00000284245
Neutrophils	FCAR	211816_x_at	2204	ENSG00000284004
Neutrophils	FCAR	211816_x_at	2204	ENSG00000284061
Neutrophils	FCAR	211307_s_at	2204	ENSG00000278415
Neutrophils	FCAR	211307_s_at	2204	ENSG00000274580
Neutrophils	FCAR	211307_s_at	2204	ENSG00000275136
Neutrophils	FCAR	211307_s_at	2204	ENSG00000275970
Neutrophils	FCAR	211307_s_at	2204	ENSG00000276985
Neutrophils	FCAR	211307_s_at	2204	ENSG00000186431
Neutrophils	FCAR	211307_s_at	2204	ENSG00000276858
Neutrophils	FCAR	211307_s_at	2204	ENSG00000275269
Neutrophils	FCAR	211307_s_at	2204	ENSG00000273738

Neutrophils	FCAR	211307_s_at	2204	ENSG00000275564
Neutrophils	FCAR	211307_s_at	2204	ENSG00000283953
Neutrophils	FCAR	211307_s_at	2204	ENSG00000283750
Neutrophils	FCAR	211307_s_at	2204	ENSG00000284245
Neutrophils	FCAR	211307_s_at	2204	ENSG00000284004
Neutrophils	FCAR	211307_s_at	2204	ENSG00000284061
Neutrophils	CEACAM3	210789_x_at	1084	ENSG00000170956
Neutrophils	FPRL1	210772_at	2358	ENSG00000171049
Neutrophils	HIST1H2BC	214455_at	8347	ENSG00000180596
Neutrophils	HPSE	219403_s_at	10855	ENSG00000173083
Neutrophils	FLJ11151	218610_s_at	55313	ENSG00000103381
Neutrophils	CREB5	205931_s_at	9586	ENSG00000146592
Neutrophils	S100A12	205863_at	6283	ENSG00000163221
Neutrophils	FCGR3B	204006_s_at	2215	ENSG00000162747
Neutrophils	TNFRSF10C	211163_s_at	8794	ENSG00000173535
Neutrophils	SLC22A4	205896_at	6583	ENSG00000197208
Neutrophils	KIAA0329	204307_at	9895	ENSG00000196663
Neutrophils	SLC25A37	218136_s_at	51312	ENSG00000147454
Neutrophils	BST1	205715_at	683	ENSG00000109743
Neutrophils	FCAR	207674_at	2204	ENSG00000278415
Neutrophils	FCAR	207674_at	2204	ENSG00000274580
Neutrophils	FCAR	207674_at	2204	ENSG00000275136
Neutrophils	FCAR	207674_at	2204	ENSG00000275970
Neutrophils	FCAR	207674_at	2204	ENSG00000276985
Neutrophils	FCAR	207674_at	2204	ENSG00000186431
Neutrophils	FCAR	207674_at	2204	ENSG00000276858
Neutrophils	FCAR	207674_at	2204	ENSG00000275269
Neutrophils	FCAR	207674_at	2204	ENSG00000273738
Neutrophils	FCAR	207674_at	2204	ENSG00000275564
Neutrophils	FCAR	207674_at	2204	ENSG00000283953
Neutrophils	FCAR	207674_at	2204	ENSG00000283750
Neutrophils	FCAR	207674_at	2204	ENSG00000284245
Neutrophils	FCAR	207674_at	2204	ENSG00000284004
Neutrophils	FCAR	207674_at	2204	ENSG00000284061
Neutrophils	CEACAM3	208052_x_at	1084	ENSG00000170956
Neutrophils	CRISPLD2	221541_at	83716	ENSG00000103196
Neutrophils	TNFRSF10C	206222_at	8794	ENSG00000173535
Neutrophils	G0S2	213524_s_at	50486	ENSG00000123689
Neutrophils	SIGLEC5	220000_at	8778	ENSG00000105501
Neutrophils	CD93	202878_s_at	22918	ENSG00000125810
Neutrophils	MGAM	206522_at	8972	ENSG00000257335
Neutrophils	MGAM	206522_at	8972	ENSG00000282607
Neutrophils	ALPL	215783_s_at	249	ENSG00000162551
Neutrophils	FPR1	205119_s_at	2357	ENSG00000171051
Neutrophils	CD93	202877_s_at	22918	ENSG00000125810
Neutrophils	PDE4B	222326_at	5142	ENSG00000184588
Neutrophils	LILRB2	210146_x_at	10288	ENSG00000274513
Neutrophils	LILRB2	210146_x_at	10288	ENSG00000131042
Neutrophils	LILRB2	210146_x_at	10288	ENSG00000275463
Neutrophils	LILRB2	210146_x_at	10288	ENSG00000277751
Neutrophils	LILRB2	210146_x_at	10288	ENSG00000276146
SW480 cancer cells	KRT5	201820_at	3852	ENSG00000186081
SW480 cancer cells	RBP1	203423_at	5947	ENSG00000114115
SW480 cancer cells	TRIM29	202504_at	23650	ENSG00000137699

SW480 cancer cells	DEFA5	207529_at	1670	ENSG00000164816
SW480 cancer cells	BMP4	211518_s_at	652	ENSG00000125378
SW480 cancer cells	EEF1A2	204540_at	1917	ENSG00000101210
SW480 cancer cells	VSNL1	203797_at	7447	ENSG00000163032
SW480 cancer cells	ASPSCR1	218908_at	79058	ENSG00000169696
SW480 cancer cells	IGF2	202409_at	3481	ENSG00000167244
SW480 cancer cells	IGF2	202409_at	3481	ENSG00000129965
SW480 cancer cells	MFAP2	203417_at	4237	ENSG00000117122
SW480 cancer cells	FGF3	214571_at	2248	ENSG00000186895
SW480 cancer cells	S100A2	204268_at	6273	ENSG00000196754
SW480 cancer cells	INHBB	205258_at	3625	ENSG00000163083
SW480 cancer cells	JAG2	209784_s_at	3714	ENSG00000184916
SW480 cancer cells	LOC89944	213713_s_at	89944	ENSG00000149328
SW480 cancer cells	BAMBI	203304_at	25805	ENSG00000095739
SW480 cancer cells	JAG2	32137_at	3714	ENSG00000184916
SW480 cancer cells	BMP7	209591_s_at	655	ENSG00000101144
SW480 cancer cells	RPP25	219143_s_at	54913	ENSG00000178718
SW480 cancer cells	RHOD	209885_at	29984	ENSG00000173156
SW480 cancer cells	DHRS2	214079_at	10202	ENSG00000100867
SW480 cancer cells	ITGB4	204989_s_at	3691	ENSG00000132470
SW480 cancer cells	NTSR1	207360_s_at	4923	ENSG00000101188
SW480 cancer cells	STRA6	221701_s_at	64220	ENSG00000137868
SW480 cancer cells	SLC1A5	208916_at	6510	ENSG00000105281
SW480 cancer cells	VSNL1	203798_s_at	7447	ENSG00000163032
SW480 cancer cells	FKBP4	200894_s_at	2288	ENSG00000004478
SW480 cancer cells	S100A3	206027_at	6274	ENSG00000188015
SW480 cancer cells	TEAD4	41037_at	7004	ENSG00000197905
SW480 cancer cells	KLK6	204733_at	5653	ENSG00000167755
SW480 cancer cells	CCND1	208711_s_at	595	ENSG00000110092
SW480 cancer cells	SLC27A5	219733_s_at	10998	ENSG00000083807
SW480 cancer cells	HOXA9	214651_s_at	3205	ENSG00000078399
SW480 cancer cells	F12	205774_at	2161	ENSG00000131187
SW480 cancer cells	LRFN4	219491_at	78999	ENSG00000173621
SW480 cancer cells	NM_024609	218678_at	10763	ENSG00000132688
SW480 cancer cells	SLC6A8	210854_x_at	6535	ENSG00000130821
SW480 cancer cells	PCTK1	207239_s_at	5127	ENSG00000102225
SW480 cancer cells	SLC6A8	213843_x_at	6535	ENSG00000130821
SW480 cancer cells	KRT13	207935_s_at	3860	ENSG00000171401
Normal mucosa	TSPAN8	203824_at	7103	ENSG00000127324
Normal mucosa	LGALS4	204272_at	3960	ENSG00000171747
Normal mucosa	LGALS4	204272_at	3960	ENSG00000282992
Normal mucosa	DCN	201893_x_at	1634	ENSG00000011465
Normal mucosa	COL3A1	215076_s_at	1281	ENSG00000168542
Normal mucosa	COL3A1	201852_x_at	1281	ENSG00000168542
Normal mucosa	CEACAM5	201884_at	1048	ENSG00000105388
Normal mucosa	TAGLN	205547_s_at	6876	ENSG00000149591
Normal mucosa	KRT20	213953_at	54474	ENSG00000263057
Normal mucosa	KRT20	213953_at	54474	ENSG00000171431
Normal mucosa	DCN	211896_s_at	1634	ENSG00000011465
Normal mucosa	MYH11	201497_x_at	4629	ENSG00000133392
Normal mucosa	MYH11	201497_x_at	4629	ENSG00000276480
Normal mucosa	FXYD3	202489_s_at	5349	ENSG00000089356
Normal mucosa	ACTG2	202274_at	72	ENSG00000163017
Normal mucosa	MYLK	202555_s_at	4638	ENSG00000065534

Normal mucosa	TPM1	210987_x_at	7168	ENSG00000140416
Normal mucosa	CDH17	209847_at	1015	ENSG00000079112
Normal mucosa	NFIB	209289_at	4781	ENSG00000147862
Normal mucosa	MGP	202291_s_at	4256	ENSG00000111341
Normal mucosa	SPARCL1	200795_at	8404	ENSG00000152583
Normal mucosa	RGS5	209071_s_at	8490	ENSG00000232995
Normal mucosa	RGS5	209071_s_at	8490	ENSG00000143248
Normal mucosa	MYH11	207961_x_at	4629	ENSG00000133392
Normal mucosa	MYH11	207961_x_at	4629	ENSG00000276480
Normal mucosa	PPAP2B	212226_s_at	8613	ENSG00000162407
Normal mucosa	COL3A1	211161_s_at	1281	ENSG00000168542
Normal mucosa	IGFBP7	201162_at	3490	ENSG00000163453
Normal mucosa	PPAP2B	209355_s_at	8613	ENSG00000162407
Normal mucosa	CALD1	201616_s_at	800	ENSG00000122786
Normal mucosa	C1R	212067_s_at	715	ENSG00000159403
Normal mucosa	CALD1	212077_at	800	ENSG00000122786
Normal mucosa	AGR2	209173_at	10551	ENSG00000106541
Normal mucosa	GNA11	564_at	2767	ENSG00000088256
Normal mucosa	HEPH	203903_s_at	9843	ENSG00000089472
Normal mucosa	GNA11	213944_x_at	2767	ENSG00000088256
Normal mucosa	GNG12	212294_at	55970	ENSG00000172380
Normal mucosa	ADH1B	209612_s_at	125	ENSG00000196616
Normal mucosa	TPM1	206116_s_at	7168	ENSG00000140416
Normal mucosa	CRIM1	202551_s_at	51232	ENSG00000277354
Normal mucosa	CRIM1	202551_s_at	51232	ENSG00000150938
Normal mucosa	FBLN1	202994_s_at	2192	ENSG00000077942
Normal mucosa	IGFBP5	211959_at	3488	ENSG00000115461
Normal mucosa	LAMA4	202202_s_at	3910	ENSG00000112769
Normal mucosa	CAV1	212097_at	857	ENSG00000105974
Normal mucosa	WASL	205809_s_at	8976	ENSG00000106299
Blood vessels	CDH5	204677_at	1003	ENSG00000179776
Lymph vessels	PDPN	204879_at	10630	ENSG00000162493
Lymph vessels	VEGFC	209946_at	7424	ENSG00000150630
Lymph vessels	FIGF	206742_at	2277	ENSG00000165197
